# AML-Related NPM Mutations Drive p53 Delocalization into the Cytoplasm with Possible Impact on p53-Dependent Stress Response

**DOI:** 10.3390/cancers13133266

**Published:** 2021-06-29

**Authors:** Aleš Holoubek, Dita Strachotová, Petra Otevřelová, Pavla Röselová, Petr Heřman, Barbora Brodská

**Affiliations:** 1 Department of Proteomics, Institute of Hematology and Blood Transfusion, U Nemocnice 1, 128 20 Prague, Czech Republic; ales.holoubek@uhkt.cz (A.H.); Petra.Otevrelova@uhkt.cz (P.O.); Pavla.Roselova@uhkt.cz (P.R.); 2 Institute of Physics, Faculty of Mathematics and Physics, Charles University, Ke Karlovu 5, 121 16 Prague, Czech Republic; dita.strachotova@mff.cuni.cz

**Keywords:** p53, nucleophosmin, mutation, acute myeloid leukemia, FLIM-FRET, Selinexor, photoconversion

## Abstract

**Simple Summary:**

Nucleophosmin (NPM) is one of the most abundant nucleolar proteins and its mutations frequently occur in acute myeloid leukemia (AML). The mutations cause aberrant cytoplasmic localization of mutated protein (NPMmut) and often mediate dislocation of NPM interaction partners. Tumor suppressor p53 is known to interact with NPM in response to genotoxic stress and its cytoplasmic localization is an unfavorable prognostic factor in cancers. This study aims to characterize the NPM-p53 interaction and to elucidate the effect of the NPM mutations on p53 localization and expression in live cells. In addition, the cellular dynamics of NPMmut and p53 after treatment with nuclear export inhibitor Selinexor is described and the mechanism of the Selinexor action proposed. Our results contribute to a better understanding of the oncogenic potential of NPM mutations.

**Abstract:**

Nucleophosmin (NPM) interaction with tumor suppressor p53 is a part of a complex interaction network and considerably affects cellular stress response. The impact of *NPM1* mutations on its interaction with p53 has not been investigated yet, although consequences of NPMmut-induced p53 export to the cytoplasm are important for understanding the oncogenic potential of these mutations. We investigated p53-NPM interaction in live HEK-293T cells by FLIM-FRET and in cell lysates by immunoprecipitation. eGFP lifetime-photoconversion was used to follow redistribution dynamics of NPMmut and p53 in Selinexor-treated cells. We confirmed the p53-NPMwt interaction in intact cells and newly documented that this interaction is not compromised by the NPM mutation causing displacement of p53 to the cytoplasm. Moreover, the interaction was not abolished for non-oligomerizing NPM variants with truncated oligomerization domain, suggesting that oligomerization is not essential for interaction of NPM forms with p53. Inhibition of the nuclear exporter XPO1 by Selinexor caused expected nuclear relocalization of both NPMmut and p53. However, significantly different return rates of these proteins indicate nontrivial mechanism of p53 and NPMmut cellular trafficking. The altered p53 regulation in cells expressing NPMmut offers improved understanding to help investigational strategies targeting these mutations.

## 1. Introduction

Nucleolus is a subnuclear compartment with multiple cellular roles, including cellular stress sensing [1]. During the stress response nucleoli become more condensed and nucleolar proteins partially translocate to the nucleoplasm. Nucleolar phosphoproteins, such as nucleophosmin (NPM) or nucleolin (NCL), participate in the stress response via complex interaction network [2]. The translocated proteins can stabilize tumor suppressor p53 either by direct interaction [3,4,5,6] or by interaction with proteins regulating intracellular p53 levels [7]. NPM is an abundant nucleolar phosphoprotein with numerous functions in important cellular processes, including the p53-dependent response to genotoxic stress [1,8]. NPM interacts with p53 as well as with other tumor suppressors, such as pRb or p14Arf [2,9,10]. While the interaction with p53 occurs via region close to the C-terminus of NPM [3], the N-terminal NPM region is responsible for NPM-oligomerization [11,12].

Ubiquitin ligase HDM2 targets the p53 for degradation by proteasome [13]. In response to stress, NPM competes with the HDM2 for binding to p53, which protects p53 from ubiquitination. Alternatively, NPM binds directly to HDM2 causing release of p53 from the ligase [14,15]. The regulatory interaction network between HDM2, p53, p14Arf and nucleolar phosphoproteins allows for rapid stabilization and activation of p53 in response to genotoxic and pathogenic stimuli [16,17]. The localization of the proteins engaged in the p53 degradation/stabilization balance is affected by their mutual interaction. The p14Arf is sequestered to nucleolus by binding to NPM, where it is kept away from p53 [18,19]. Protein p53 was also observed to be pulled to the nucleolus by overexpressed NPM [3]. As another nucleolar phosphoprotein NCL does not possess nucleolar localization sequence (NoLS) [20,21], its nucleolar localization is supposed to be mediated by its association to NPM [21]. Independently of NoLS, attachment of NPM to the nucleolus has been ascribed to the affinity of its C-terminal region to G-rich nucleic acids [22].

To our knowledge, any systematic investigation of the p53-NPM interaction in cells under conditions other than the genotoxic stress has not been performed yet. In this work we therefore focus on the p53-NPM interaction in intact cells under physiological conditions. Protein tagging by fluorescent proteins makes possible monitoring of the cellular dynamics and protein proximity directly in live cells [23,24,25]. Fluorescence lifetime imaging (FLIM) utilizing resonance energy transfer (FRET) between the tagged proteins is a highly sensitive tool for this purpose [26,27]. We characterized p53-NPM interaction both in cell lysates and live cells using methods previously established for the detection of NPM oligomers, i.e., fluorescence confocal microscopy [28], FLIM-FRET [26] and immunochemical methods [27].

A mutation of the *NPM1* gene resulting in the altered C-terminus of NPM and aberrant localization of mutated NPM to the cytoplasm appears in approximately 50% AML with normal karyotype [10,29,30]. Leukemogenic potential of the mutation has not been elucidated yet. When it occurs as an isolated mutation without concurrent genetic aberrations, it stratifies the patient to the low-risk category [31]. Moreover, as refractory mutation, it is suitable for assessment of minimal residual disease (MRD) [32,33]. The original NoLS of wild-type NPM (NPMwt) is highly compromised in the mutated protein and strong nuclear export signal (NES) for the XPO1 exporter appears at the altered C-terminus [34,35] in addition to the two NESes already present in its N-terminal domain [12]. The most frequent AML-related mutation type A gives rise to mutated protein lacking tryptophans W288 and W290 (NPMmutA, further abbreviated NPMmut) [36]. An alternative mutation of type E retains W288, which partially preserves nucleolar localization of the mutated protein (NPMmutE) [37]. The interaction of NPM monomers within the oligomer and its interaction with p14Arf were shown to persist in presence of the NPMmut [34]. Interacting proteins NPMwt and p14Arf become partially dislocated to the cytoplasm due to their binding to NPMmut [38]. In analogy, other NPM-interacting proteins, e.g., p53, are also candidates for such dislocation. The dislocation should interfere with their proapoptotic activity, which could lead to uncontrolled cell division [11]. On the other hand, the interaction of NPM with NCL, taking place through AA187-241 region of the NPM molecule [21], is inhibited by the NPM mutation and NCL is therefore not translocated to the cytoplasm together with NPMmut [28]. Since p53 was found to interact with a domain near the C-terminus of NPM (AA186-259 or AA242-269, respectively) [3,4], one could expect that the p53-NPM interaction was affected by this mutation as well. The detailed mechanism and role of the p53-NPMmut interaction in the leukemogenesis is unknown so far. The main part of this article therefore investigates impact of the NPM mutation on the p53-NPM interaction. This interaction is confirmed in cell lysates, and we also newly document it in live cells. Importantly, we bring evidence that the mutation has no impact on this interaction.

Cytoplasmic localization of p53 plays an adverse role in the cell cycle regulation [39] and it was reported to launch apoptosis via interaction with mitochondrial proapoptotic factors [40]. On the other hand, displacement of the p53 from its mainly nuclear localization could lead to aberrant growth signals resulting in cancer development [41]. The proteasome-mediated p53 degradation can also be affected by the p53 translocation [42]. Therapeutic restoration of the p53 functionality therefore seems to be a promising anti-cancer approach [43]. As NPM and p53 mutations are mutually exclusive in AML, the return of wild type p53 to the nucleus could re-establish its tumor suppressor and cell cycle inhibitor function. The blockade of exportin 1 (XPO1) is a way how to ensure it [44].

The XPO1 mediates outward protein transport from the nucleus [45]. The transport efficiency depends on the recognition of specific leucine-rich NESes [46]. Two proper NESes were identified in p53 [47,48]. Leptomycin B is a typical drug blocking XPO1. As this drug is too toxic for the clinical use [49], selective inhibitors of the nuclear export (SINE) attract attention nowadays [50]. These drugs, such as Selinexor, were reported to reactivate p53 by restoring its nuclear accumulation via the XPO1 blockade and, consequently, its proper regulatory function [41]. Since NPMmut also binds to XPO1, it is not surprising that Leptomycin B or Selinexor have been reported to induce relocalization of NPMmut to the nucleus [36,51]. Selinexor has already been tested both for treatment of *TP53*- and *NPM1*- mutated cancers with modest results [52]. In the second part of this work, we address p53 interactions and trafficking in HEK-293T cells and leukemia cell lines treated with Selinexor in order to elucidate mechanism of p53 and NPMmut co-translocation in live cells. Irrespective to clinical applicability of Selinexor, obtained data offer deeper understanding of the involved processes to help investigational strategies searching for more targeted treatments.

## 2. Materials and Methods

### 2.1. Cell Cultivation

Adherent cell line HEK-293T was kindly provided by dr. Šárka Němečková (Department of Immunology, Institute of Hematology and Blood Transfusion). Leukemia cell lines MV4-11, OCI-AML2, OCI-AML3 and KASUMI-1 were purchased from DSMZ (Braunschweig, Germany). The cells were cultivated in growth media with fetal bovine serum (FBS), glutamine and antibiotics (all from Sigma-Aldrich, Darmstadt, Germany) according to manufacturers’ recommendation: MV4-11 in RPMI-1640/10% FBS, OCI-AML2 and OCI-AML3 in alpha-MEM/20% FBS, KASUMI-1 in RPMI-1640/20% FBS and HEK-293T in DMEM/10% FBS. Selinexor (Selleckchem, Houston, TX, USA) was added into the cell culture from 10 mM stock solution to final concentrations and for times specified in the text.

### 2.2. Plasmid Construction and Cell Transfection

Plasmids for expression of fluorescently tagged proteins were constructed by standard molecular cloning techniques. Preparation of plasmids for NPMwt and NPMmutA expression was described in [53], for NPMmutE expression was described in [35] and for Δ117wt in [27]. For cloning, DNA fragments corresponding to specific *NPM1* and *TP53* transcript variants (RefSeq. NM 002520.7, https://www.ncbi.nlm.nih.gov/nuccore/NM_002520.7 and NM 000546.6, https://www.ncbi.nlm.nih.gov/nuccore/NM_000546.6, respectively, from NCBI database accessed on 25 April 2021) were PCR-amplified from cDNA library (Jurkat cells, OriGene, Rockville, MD, USA) or from plasmid pBI-p53 wt/EGFP (Plasmid #16543, Addgene, http://n2t.net/addgene:16543; RRID: Addgene_16543, accessed on 7 June 2021) using extended primers and subsequently they were subcloned to vectors peGFP-C2 or pmRFP1-C2 (originally Clontech, Saint-German-en-Laye, France) using XhoI and BamHI unique restriction sites (Thermo Scientific, Waltham, MA, USA) and T4 DNA ligase (NEB, Ipswich, MA, USA). Plasmids for Δ117mut bearing mutation type A were prepared according to [27,53] by combination of primers used for construction of the single mutants to obtain the desired double mutant. The pBI-p53 wt/EGFP plasmid was a gift from Bert Vogelstein (Addgene plasmid # 16543) [17].

The constructed plasmids were amplified in *E. coli* competent cells and purified with the PureYield Plasmid Miniprep System (Promega, Madison, WI, USA). HEK-293T cells were seeded to the cell density of 1 × 10^5^/mL 24 h prior transfection and then transfected with jetPrime transfection reagent (Polyplus transfection, Illkirch, France) according to the manufacturer’s protocol. Growth medium was replaced 4 h after the transfection and cells were further grown for 20–40 h prior analysis. NPM and p53 constructs appearing in this study are listed in Table 1.

### 2.3. Cell Lysis

As described previously [28], cells were washed with PBS and lysed in Laemmli sample buffer (SB, 50 mM Tris pH 6.8, 2% SDS, 100 mM DTT, 10% glycerol), boiled at 95 °C for 5 min, centrifuged at 200,000× *g*/4 °C for 4 h and the supernatant was stored at −20 °C.

### 2.4. Western Blotting

Five to ten microliters of each sample were subjected to SDS-PAGE and transferred into PVDF membrane (BioRad, Hercules, CA, USA). Mouse monoclonal antibodies against β-actin, GFP, dsRed, NCL, FBL and NPM (clone 3F291, for endogenous NPM), were from Santa Cruz Biotechnology (Dallas, TX, USA). All mouse primary antibodies were used at a dilution 1:100–1:500 (see Table S1 for details). Rabbit monoclonal antibody against p53 (Abcam, Cambridge, U.K.) and rabbit polyclonal antibodies against NPM (Abcam) and NPMmut (pab50321, Covalab, Bron, France) were used at 1:1000–1:2000 dilution (see Table S1 for details). Primary antibodies used for Supplementary Figure S6 are specified in Table S2. Anti-mouse and anti-rabbit HRP-conjugated secondary antibodies were purchased from Thermo Scientific (Waltham, MA, USA) and used at concentrations 1:50,000, or 1:20,000 for the detection of co-precipitated proteins. ECL Plus Western Blotting Detection System (GE Healthcare, Chicago, IL, USA) was used for chemiluminescence visualization and evaluation by G-box iChemi XT4 digital imaging device (Syngene Europe, Cambridge, U.K.). Alternatively, SuperSignal West Atto Ultimate Sensitivity Substrate (ThermoFisher Sci, Waltham, MA, USA) was used for highly sensitive detection of co-immunoprecipitated proteins. Presented data are always representative of at least three independent experiments.

### 2.5. Co-Immunoprecipitation Trap Assay

Transfected cells expressing fluorescent proteins were processed after 40 h-incubation. GFP-, RFP- and p53-Trap_A systems (Chromotek, Planegg, Germany) were used following the manufacturer’s instructions as described in [35]. Briefly, eGFP/mRFP1-expressing adherent cells were washed with in ice-cold PBS and scrapped from dish. Alternatively, suspension cells of leukemia cell lines were collected by centrifugation and washed with PBS. The cell pellet was lysed in the lysis buffer (10 mM Tris/Cl pH 7.5, 150 mM NaCl, 0.5 mM EDTA, 0.5% NP-40, protease and phosphatase inhibitors) on ice for 30 min and centrifuged at 20,000× *g*/10 min/4 °C. The lysate was applied on the Trap_A beads and rotated for 1 h at 4 °C. Then the beads were pelleted and extensively washed in the diluting buffer (10 mM Tris/Cl pH 7.5, 150 mM NaCl, 0.5 mM EDTA), resuspended in 2XSDS-sample buffer (100 mM Tris pH 6.8, 4% SDS, 200 mM DTT, 20% glycerol), boiled for 10 min and centrifuged at 2500× *g*/2 min/4 °C. Supernatant was stored at −20 °C until used for SDS-PAGE.

### 2.6. Live Cell Imaging

Cells were grown on glass bottom Petri dish (Cellvis, Mountain View, CA, USA). Fluorescence experiments were carried out at 37 °C after sealing the Petri dish with parafilm to prevent CO_2_ leakage. The subcellular distribution and co-localization of eGFP- and mRFP1-fused NPM variants were observed under the confocal laser scanning microscope FV1000 (Olympus Corporation, Tokyo, Japan). One Petri dish was typically imaged for 1 h and data were processed by FluoView FV10-ASW 3.1 (Olympus, Tokyo, Japan) and ImageJ-FiJi software (FiJi software, Bethesda, USA).

### 2.7. FLIM—Data Acquisition and Analysis

FLIM experiments were carried out on the inverted IX83 microscope with FV1200 confocal scanner (Olympus, Tokyo, Japan). The microscope was equipped with cell-cultivation chamber (Okolab, Pozzuoli, NA, Italy) and FLIM add-on comprising picosecond semiconductor lasers, fiber-coupled GaAsP hybrid detectors and TimeHarp 260PICO TCSPC detection electronics (all PicoQuant, Berlin, Germany). Specifically, cellular FLIM experiments were performed with a UPLSAPO 60× NA 1.2 water immersion objective (Olympus), eGFP fluorescence was excited at 485 nm by the LDH-DC-485 laser head (PicoQuant) and emission decays were collected on the pixel-by-pixel basis using combination of 560 nm short-pass dichroic and Semrock 520/35 bandpass filter in the detection path. To avoid pile-up, the data collection rate at brightest pixels was kept below 5% of the laser repetition rate. Acceptor photobleaching was done by the 562 nm cw laser. eGFP lifetime-photoconversion (PC) was performed as described previously [55]. Selected ROI was scanned by intense 488 nm light till eGFP emission decreased to about 20–30% of its initial value. Depending on the sample, the power of the argon 488 nm cw-laser line was set between 0.5 and 1.0 mW at the back-aperture of the objective in order to complete the lifetime-conversion within 1–2 min. All experiments were performed at 37 °C.

FLIM data were analyzed using the SymPhoTime64 software (PicoQuant, Berlin, Germany). The lifetime images were generated in the SymPhoTime64 by the “fast-FLIM” approach when mean pixel lifetimes were calculated by a method of moments [56]. Specifically, the lifetime *τ_fast_* was determined as the difference between the barycentre of the fluorescence decay and the time-offset *t_offset_* at the steepest growth of the decay curve at each pixel:

(1) $$\tau_{fast}=\frac{\sum I_{i}t_{i}}{\sum I_{i}}-t_{offset}$$

where *I_i_* stands for the emission intensity at time *t_i_*. Least-squares reconvolution was applied for accurate analysis of cumulative decays from larger ROIs (cell, nucleus, nucleolus). Fluorescence of eGFP was typically assumed to decay bi-exponentially [57,58] according to the formula:

(2) $$I\left(t\right)=a_{1}\cdot e^{-t/\tau_{1}}+a_{2}\cdot e^{-t/\tau_{2}}$$

where *τ_i_* and *a_i_* are lifetime components and corresponding amplitudes, respectively. The intensity-weighted mean fluorescence lifetime was calculated as:

(3) $$\tau_{mean}=\sum f_{i}\cdot \tau_{i},\;f_{i}=a_{i}\tau_{i}/\sum a_{i}\tau_{i}$$

where *f_i_* are intensity fractions of the *i*-th lifetime component.

## 3. Results

**Scheme 1:**
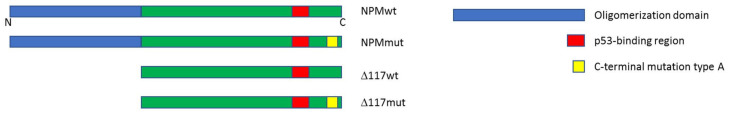
Comparison of used NPM variants. List of abbreviations is in Table 1.

We investigated interaction of fluorescently labeled p53 with NPM variants described in Scheme 1. Subcellular localization of the studied proteins after the transfection into HEK-293T cells is shown in Figure S1.

### 3.1. Interaction of p53 with NPMwt and NPMmut

**Figure 1:**
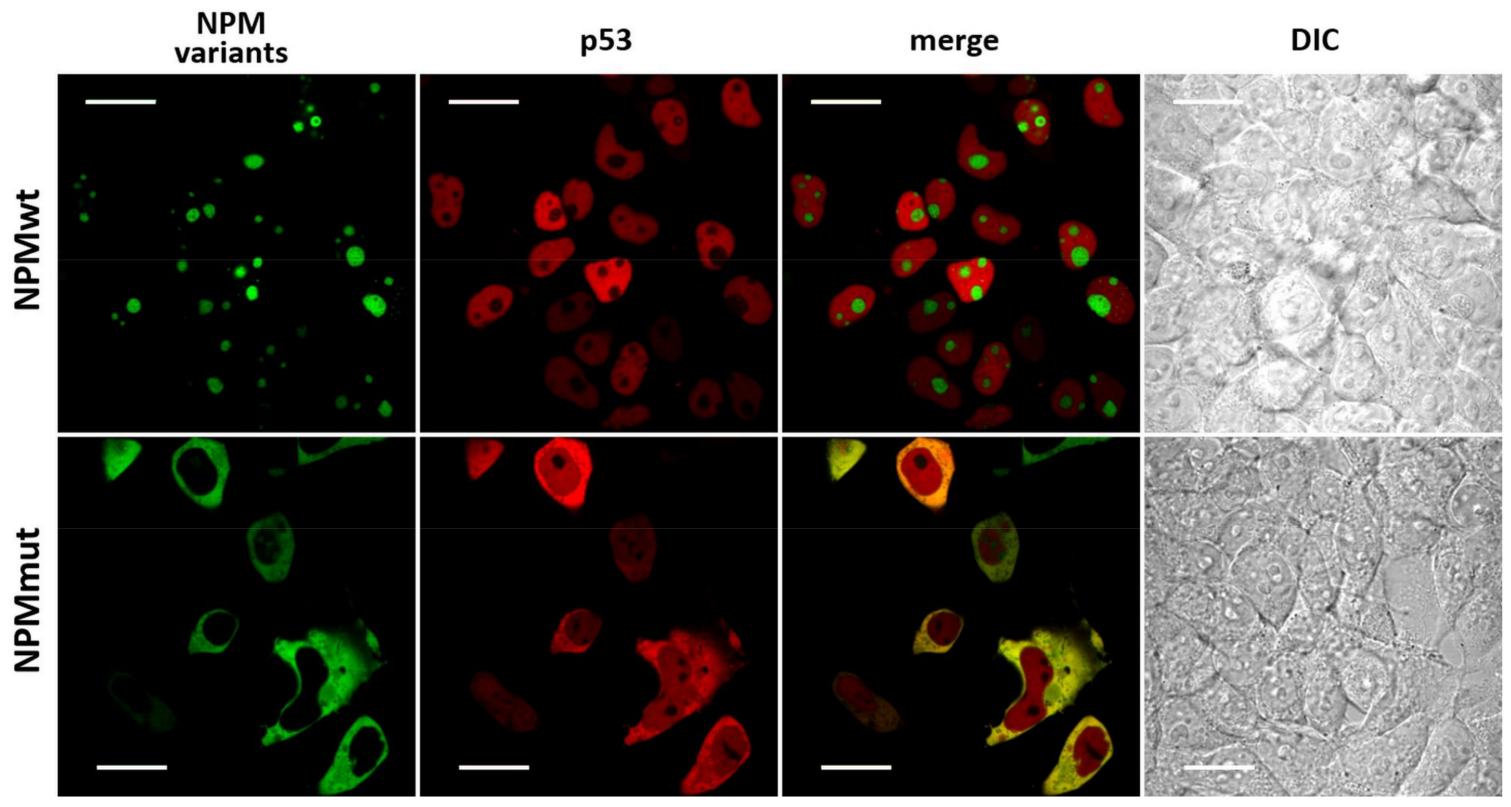
Localization of R_p53 co-expressed with eGFP-labeled NPM variants in HEK-293T cells. Upper row—R_p53/G_NPMwt co-expression, lower row—R_p53/G_NPMmut co-expression, first column—eGFP fluorescence, second column—mRFP1 fluorescence. Bar indicates 20 µm, abbreviations can be found in Table 1.

**Figure 2:**
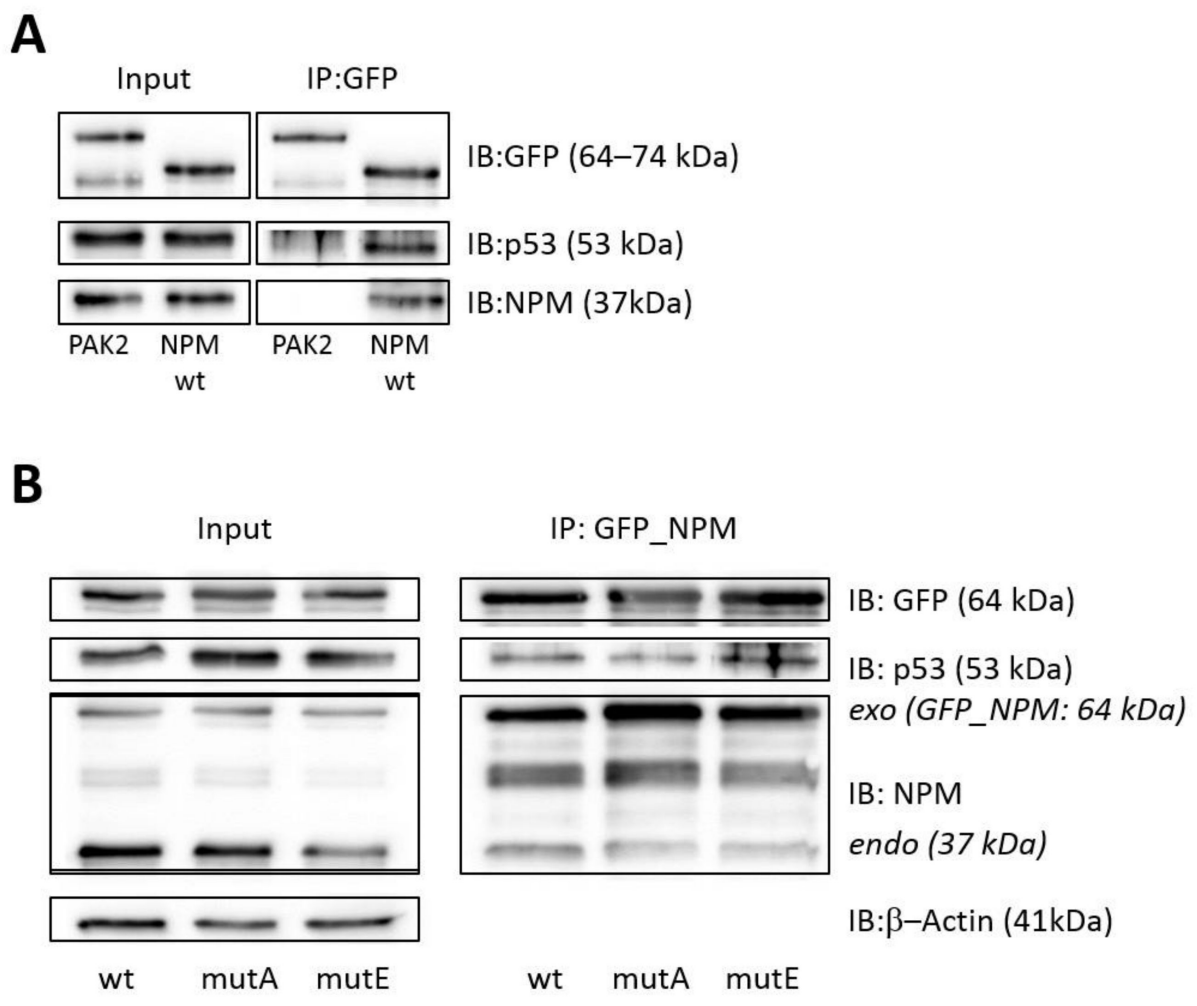
Interaction of p53 with NPM variants in lysates of HEK-293T cells. (**A**) Interaction of p53 with NPMwt. Cells were transfected with G_NPMwt or G_PAK2 (p53-non-interacting control of a comparable size). Endogenous NPM as well as p53 co-precipitated only with NPMwt; (**B**) interaction of p53 with NPMmut. Cells were transfected with eGFP-labeled NPMmutA, NPMmutE or NPMwt for comparison (see Methods, Section 2.2). Endogenous NPM, as well as p53, co-precipitated in all samples. The samples are identical with those used in [28] for assessment of the interaction between NPM and nucleolin. Reprinted with permission from Ref. [28]. Copyright Elsevier (2018). Abbreviations can be found in Table 1.

The C-terminal mutation drives NPM translocation to the cytoplasm. Here we investigated interaction of fluorescently tagged NPMwt and NPMmut with p53 in transfected HEK-293T cells, similarly presented for NPM oligomers in [27,28]. As seen in Figure 1, exogenous p53 is markedly delocalized to the cytoplasm of HEK-293T cells co-expressing NPMmut. We observed extensive translocation of mRFP1_p53 (R_p53) to the cytoplasm when co-expressed with eGFP-tagged NPMmut (G_NPMmut). This is in the clear contrast with p53 localized mainly in the nuclei in single p53-transfected cells (Figure S1) or in cells co-expressing eGFP-tagged NPMwt (G_NPMwt). Although the effect of co-expression on the p53 translocation is clearly observable in both tagging combinations, Figure S2, it is more obvious for the G_NPMmut/R_p53 combination. The observed translocation suggests that p53 interacts with NPM regardless the C-terminus mutation. We confirmed this finding by co-immunoprecipitation as done for endogenous NPM in mixed NPM oligomers [27]. Specifically, NPMwt and NPMmut were labeled with eGFP, transfected into HEK-293T cells and immunoprecipitated with the GFP-Trap. Then co-immunoprecipitated endogenous p53 was detected by immunoblotting. Indeed, the interaction between p53 and NPMwt, as well as between p53 and NPMmut was proven, Figure 2. The endogenous p53 was clearly detected in all precipitates, regardless NPM mutation type. Supplementary Figure S3 shows results of alternative experiments, which document the interactions using p53-Trap for the endogenous p53 immunoprecipitation and combination of GFP/RFP Traps for immunoprecipitation from cells co-expressing R_NPMwt and G_p53. These results bring further evidence for our new finding that the p53-NPM interaction is not inhibited by the mutation of the NPM C-terminus.

### 3.2. Effect of the NPM Oligomerization Domain

**Figure 3:**
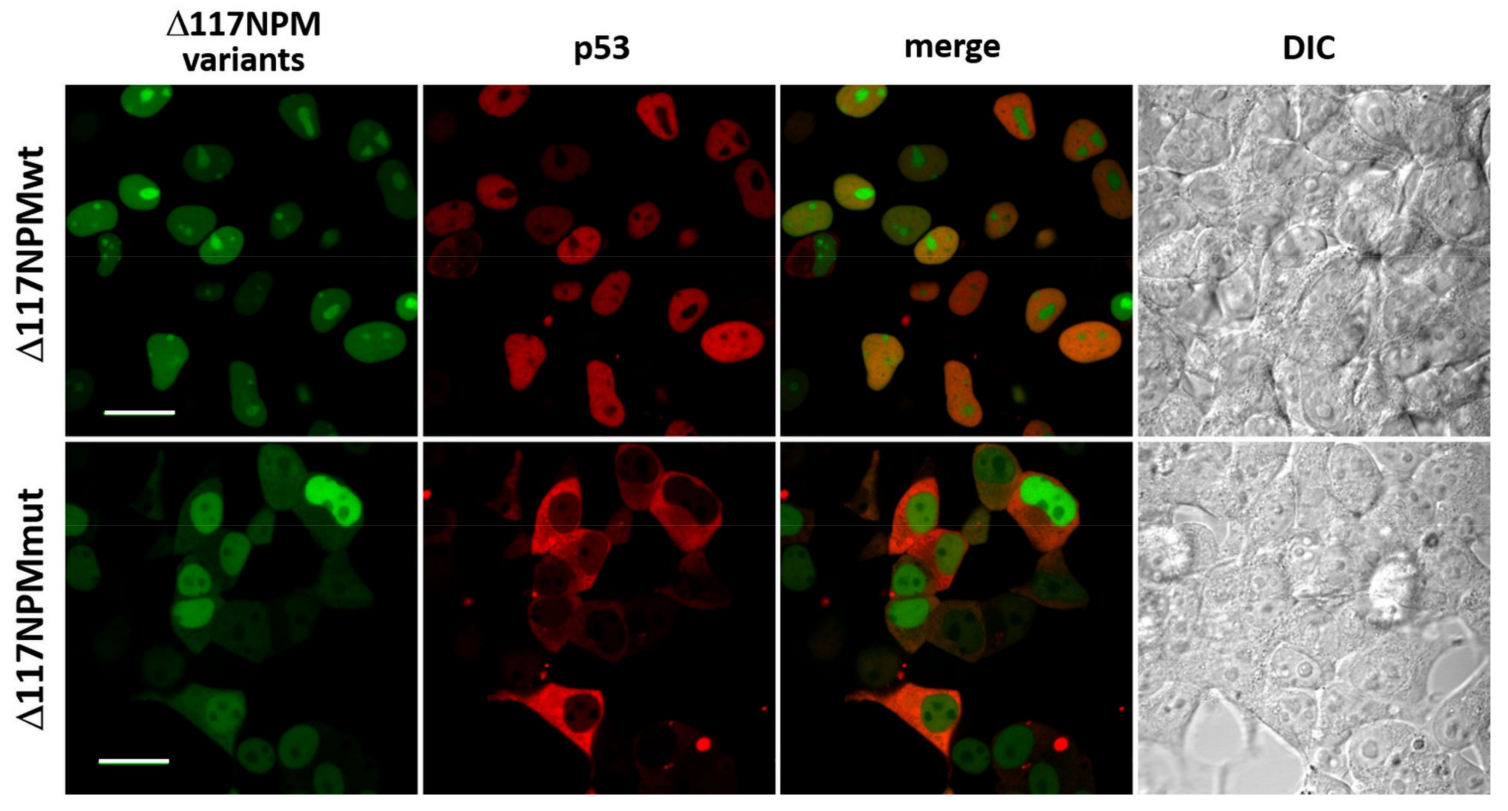
Localization of R_p53 co-expressed with eGFP-labeled NPM variants with truncated oligomerization N-terminus (AA1-117) in HEK-293T cells. Upper row—R_p53/G_Δ117wt co-expression, lower row—R_p53/G_Δ117mut co-expression, first column—eGFP fluorescence, second column—mRFP1 fluorescence. Bar indicates 20 µm, abbreviations can be found in Table 1.

**Figure 4:**
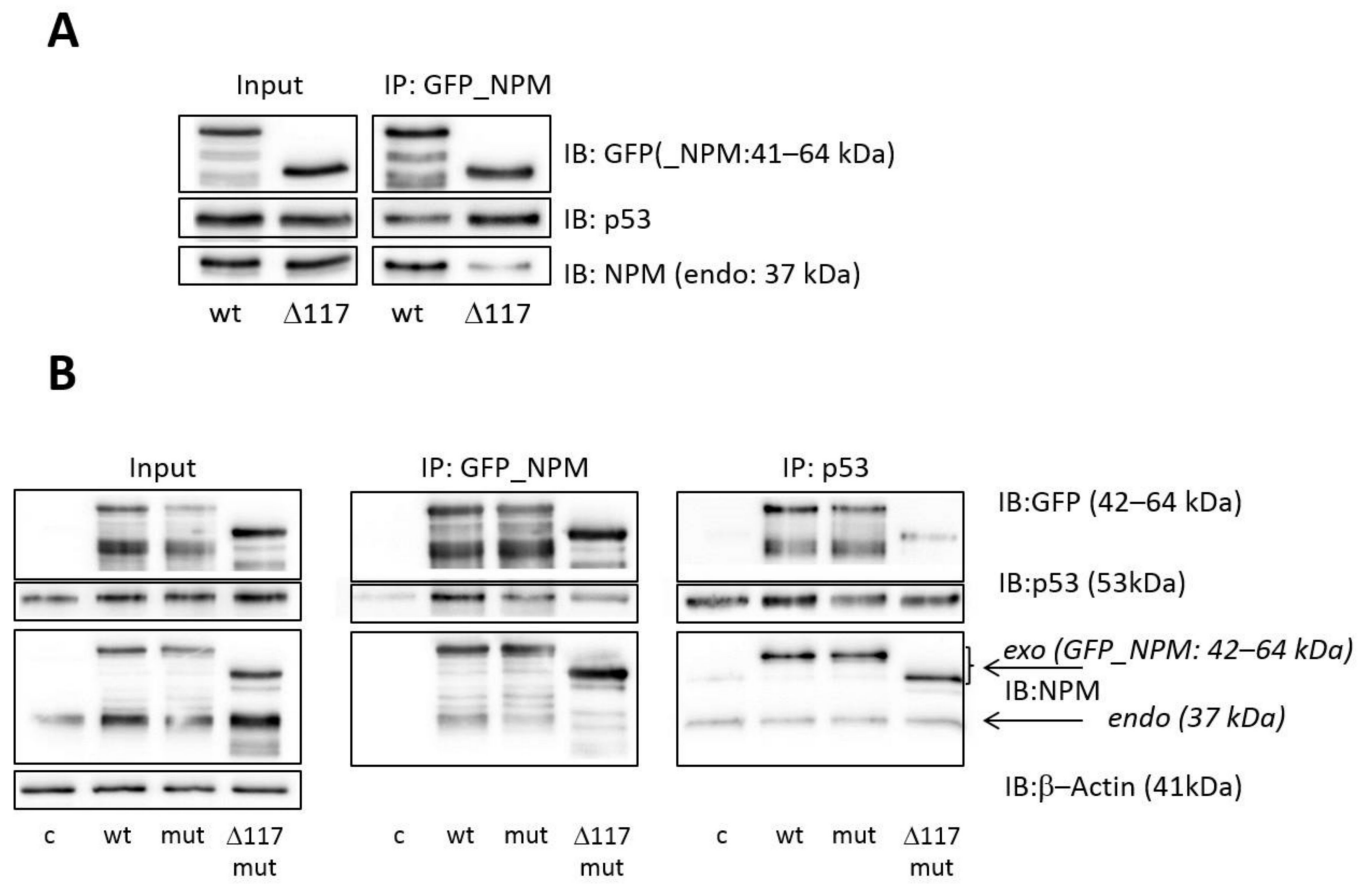
Interaction of p53 with Δ117 variants of NPM in lysates of HEK-293T cells. (**A**) Interaction of p53 with Δ117wt. Cells were transfected with G_Δ117wt or G_NPMwt for control. Levels of co-precipitated endogenous NPM and p53 differ between the samples; (**B**) interaction of p53 with Δ117mut. Cells were transfected with G_NPMmut, G_Δ117mut or G_NPMwt for comparison. Untransfected cells (c) served as a precipitation control. Levels of co-precipitated proteins were investigated by immunoblotting. β-Actin expression served as a loading control. Abbreviations can be found in Table 1.

Further we investigated the role of NPM oligomerization in the p53 delocalization and NPM-p53 interaction. As the oligomerization of NPM is mediated by its N-terminal domain (AA1-117) [59,60], we truncated the N-terminus to obtain NPMwt and NPMmut variants with compromised oligomerization, Δ117wt [27] and Δ117mut, respectively, Figure 3. Similar to Δ117wt, we found Δ117mut being localized mainly in the nucleus, nevertheless, with substantial cytoplasmic fraction caused by the C-terminal mutation (Figure S1). At the same time, extensive translocation of R_p53 to the cytoplasm was observed when co-expressed with eGFP-tagged Δ117mut (G_Δ117mut). The observation strongly suggests an existence of interaction between p53 and Δ117mut, similar as for NPMmut. The effect of p53 co-expression with Δ117mut on the p53 translocation to the cytoplasm is comparable in both tagging combinations, Figure S4. To independently confirm the p53-Δ117mut interaction, we performed immunoprecipitation experiments presented in Figure 4. Consistently with our previous results [27], we detected slightly weaker interaction between NPMwt and Δ117wt than between NPMwt monomers. Concurrently, endogenous p53 was pulled down with Δ117wt more efficiently than with NPMwt, suggesting that the interaction between p53 and Δ117wt might be even stronger than between p53 and NPMwt (Figure 4A). In Δ117mut precipitates the endogenous p53 was detected as well, but in a lower extent, Figure 4B. Endogenous NPMwt did not precipitate with Δ117mut at all, which indicates inhibited oligomerization of this construct. Presented results document that the interaction capability of NPMwt with p53 is independent of the NPM oligomerization status, which implicates that monomeric NPM is able to interact with p53. However, the lower extent of exogenous p53 detected in Δ117mut precipitates indicates lower binding affinity for the double mutant.

### 3.3. Interaction of p53 and NPM in Live Cells

**Figure 5:**
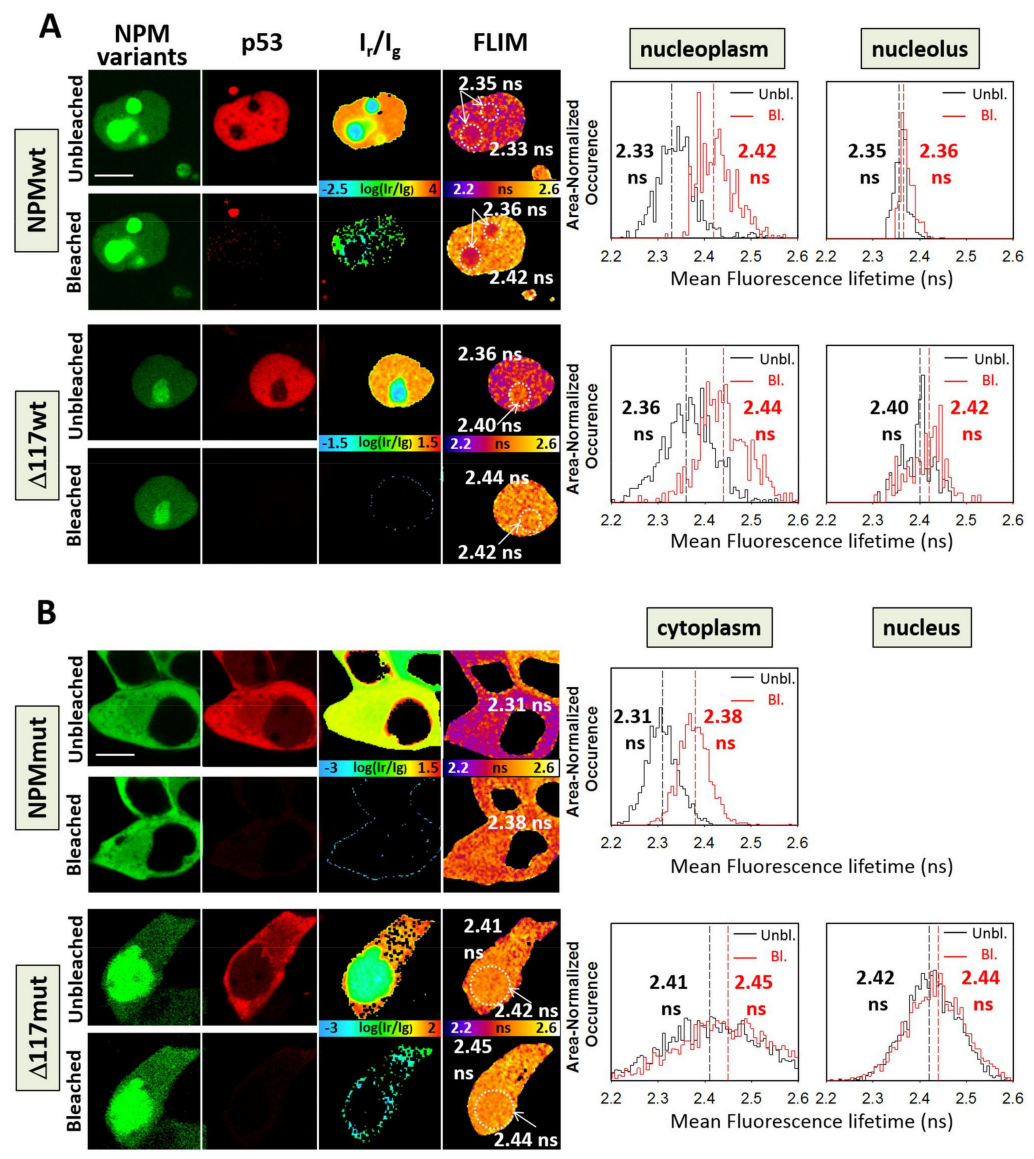
Interaction of R_p53 with eGFP-labeled variants of NPM in live HEK-293T cells examined by FLIM-FRET. (**A**) Interaction of p53 with NPM variants with unmodified C-terminus. Upper two rows—co-expression of R_p53 with G_NPMwt, lower two rows—co-expression of R_p53 with G_Δ117wt; (**B**) interaction of p53 with NPM variants bearing mutation at the C-terminus. Upper two rows—co-expression of R_p53 with NPMmut, lower two rows—co-expression of R_p53 with G_Δ117mut. Columns from left to right represent localization of the green fluorescence I_green_ (NPM variants), red fluorescence I_red_ (R_p53), ratio I_red_/I_green_, and fluorescence lifetime maps of eGFP before (row Unbleached) and after (row Bleached) mRFP1 photobleaching. Histograms contain pixel-based lifetime distributions from the nucleolus and nucleoplasm before (black line) and after (red line) the mRFP1 photobleaching. Bar represents 10 µm, abbreviations can be found in Table 1. Representative results from 3–5 repeated experiments are shown.

To investigate the p53-NPMmut interaction in live cells we utilized FLIM-FRET, which visualizes fluorescence lifetime across the cellular structures. FRET reliably detects proximity of the donor and the acceptor on the molecular scale and presents itself by shortening of the donor fluorescence lifetime [61,62]. eGFP and mRFP1 fused to p53 and NPM were used as the donor and the acceptor, respectively (the reverse labeling was used where specified, see Table 1). The shortening of the eGFP lifetime (τ_GFP_) in the presence of the acceptor-labeled protein then indicates molecular complex formation. To unambiguously prove the presence of the energy transfer, the acceptor was photobleached, which cancels FRET. Consequently, τ_GFP_ should increase [63]. Interaction of R_p53 with G_NPM variants is presented in Figure 5. Images from experiment with the G_NPMwt/R_p53 combination are presented in the first two rows of Figure 5A. Presence of FRET between G_NPMwt and R_p53 is clearly documented in the nucleoplasm by the increase of τ_GFP_ from 2.33 to 2.42 ns upon the acceptor photodestruction. Nucleoli without sufficient concentration of the acceptor serve as an alternative internal control and FRET does not occur there, as expected. R_p53 co-expressed with G_Δ117wt displays very similar behavior (Figure 5A, lower two rows). Compared to the mainly nucleolar G_NPMwt [27], the amount of G_Δ117wt in the nucleoplasm is enhanced and FRET between G_Δ117wt and R_p53 is clearly present there, as τ_GFP_ increases from 2.36 to 2.44 ns upon the acceptor photobleaching. Nucleolus with a low acceptor concentration serves again as the internal control and FRET is not detected there. Lifetime histograms of the G_NPMwt–R_p53 and G_Δ117wt–R_p53 pairs highly resemble each other indicating similar interaction. In agreement with the immunostaining, we proved that the lack of the NPM oligomerization domain does not evidently prevent the interaction between p53 and NPM monomers.

Interaction between G_NPMmut and R_p53 is assessed in the first two rows of Figure 5B. FRET was detected in the cytoplasm where the donor lifetime increases from 2.31 ns to 2.38 ns after the acceptor destruction. In agreement with Figure 1, G_NPMmut is almost absent in the nuclei, which prevents FRET measurements there. The last two rows in Figure 5B document the cellular interaction between R_p53 and G_Δ117mut. We can see that R_p53 is strongly delocalized to the cytoplasm while the nucleus is depleted. The opposite is true for G_Δ117mut since the lack of NESes from oligomerization domain suppresses cytoplasmic transport of Δ117mut relatively to NPMmut and the protein stays mainly in the nucleus. In contrast to the immunostaining, we were not able to prove FRET between G_Δ117mut and R_p53 neither in the cytoplasm nor in the nucleus of live cells. As R_p53 is translocated to the cytoplasm, there is not enough acceptor in the nucleus to observe FRET, which is clearly visible in a lifetime histogram in the last column of Figure 5B. Although the acceptor is in a significant excess in the cytoplasm and FRET should be detected, lifetime histograms show only minor change there before and after the photobleaching. The observed discrepancy might be explained by rather low binding affinity of p53 to G_Δ117mut, as suggested by the low amount of endogenous p53 co-immunoprecipitating with G_Δ117mut, compared to G_NPMmut and G_Δ117wt (Figure 4B). Binding conditions and concentrations in live cells might be therefore unfavorable for the detection of trace amounts of the complex by FRET. Alternatively, the critical Forster distance could be somehow larger for this particular protein combination, e.g., due to an unfavorable orientation factor of the labels [64]. The weak interaction then might be missed by FRET. The intriguing observation shown in the last two columns of Figure 5 indicates a nontrivial mechanism of NPM/p53 cellular co-trafficking when both NPM termini are altered.

Summary of the results obtained by confocal microscopy and immunoprecipitation is shown in Table 2.

### 3.4. Selinexor-Induced p53 and NPM Relocalization

**Figure 6:**
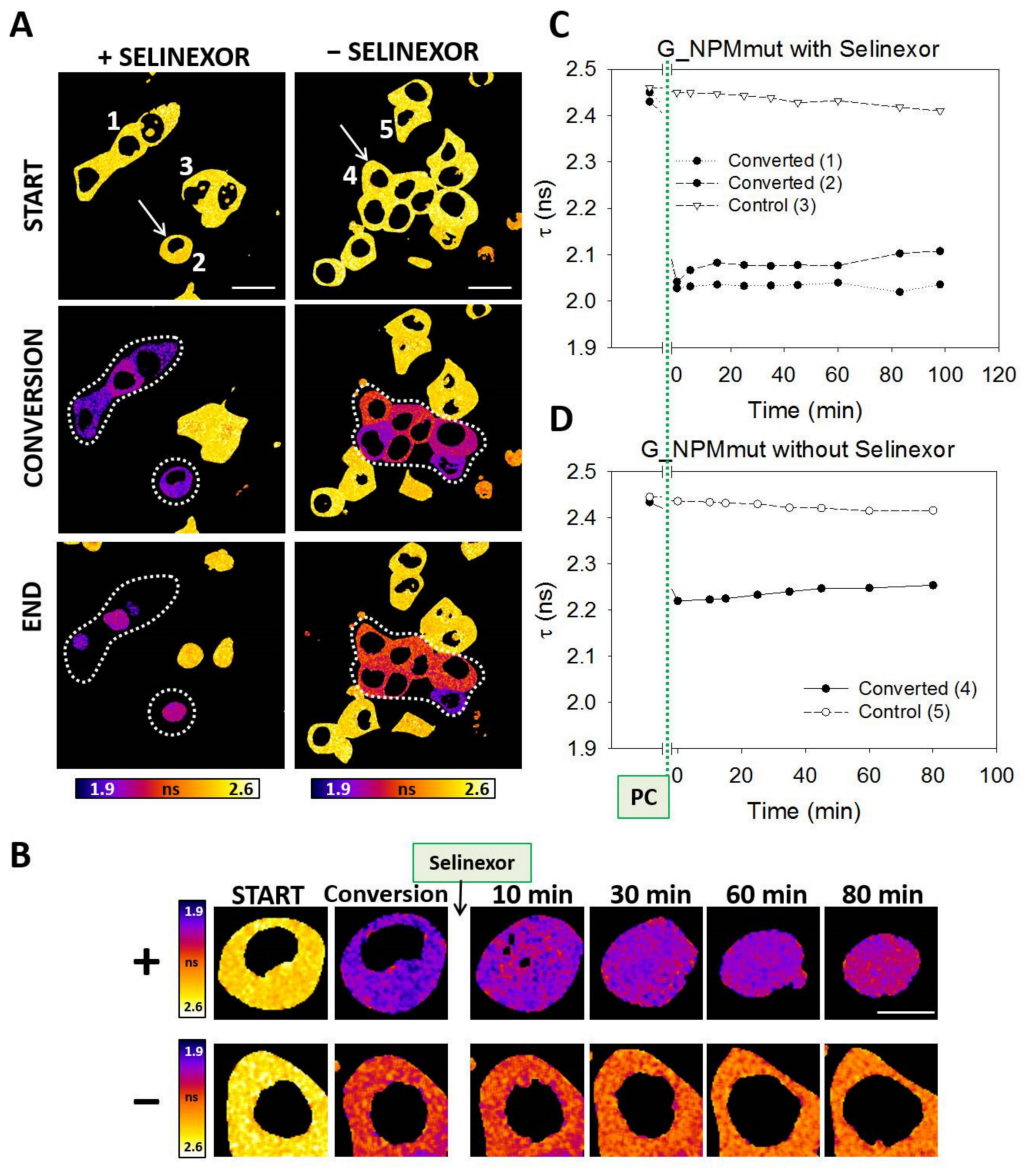
Effect of Selinexor on localization and lifetime of G_NPMmut in live HEK-293T cells. (**A**) Lifetime images of G_NPMmut in the presence and absence of 4 µM Selinexor before (the 1st row), immediately after (the 2nd row) and 80 min after the photoconversion (PC) (the 3rd row). The photoconverted area is bordered by the dashed line. Bar represents 20 µm; (**B**) detailed time evolution of G_NPMmut lifetime maps in single cell with photoconverted eGFP in the presence (upper row, cell no. 2) and in the absence (lower row, cell no. 4) of Selinexor. The visualized cells are marked by arrows in panel A. The nuclear signal was negligible in the absence of Selinexor. A minute increase in τ_GFP_ is noticeable both in the presence and absence of Selinexor. Bar represents 10 µm; (**C**,**D**) time evolution of τ_GFP_ in the photoconverted cells (1, 2, 4) and in control cells (3, 5) in the presence (**C**) and absence (**D**) of Selinexor. Cells used for integration of the plotted signal are marked in the panel A. Abbreviations can be found in Table 1. Representative results from 3–5 repeated experiments are shown.

Further we investigated effect of Selinexor on the p53 and NPMmut relocalization back to the nucleus. Since both NPM and p53 bind to XPO1, we tried to verify whether their Selinexor-induced nuclear import is synchronous in a complex, as could be assumed from the p53-NPMmut interaction. To monitor the relocalization, we performed time-lapse FLIM measurement utilizing the lifetime-based photoconversion (PC) of eGFP [55]. The method allows marking a selected population of eGFP-labeled protein by a brief scan by intense 488 nm light, which irreversibly converts eGFP to a form with a shorter emission lifetime. Since the photoconverted and unconverted tags differ in the lifetimes, diffusion and cellular redistribution of the highlighted protein can be easily followed by FLIM. This allows quantification of the converted and unconverted eGFP fractions across the cell by the localized lifetime analysis. First, we monitored the effect of Selinexor on the relocalization of G_NPMmut (Figure 6). After the PC, the FLIM image was measured and then 4 µM Selinexor was added. Rows of Figure 6A from the top to bottom show FLIM images before the PC, just after the PC and 80 min later. The photoconverted areas are marked by the dashed line. We can notice significant difference in the G_NPMmut localization after the Selinexor treatment (first column). While G_NPMmut stays in the cytoplasm in the control sample (second column), the addition of Selinexor induced marked relocalization of G_NPMmut to the nucleus that was finished in about 60 min. Figure 6B demonstrates this process in more detail for selected cells in the presence and the absence of Selinexor, cell no. 2 and 4, respectively (panel A). Quantitative evolution of mean eGFP lifetimes (τ_GFP_) in individual cells is depicted in Figure 6C,D. It can be immediately seen that the addition of Selinexor has negligible effect on the time evolution of τ_GFP_ of eGFP tagging NPMmut either in the control or in the photoconverted cells (compare differences between panels C and D). The small rise in τ_GFP_ in the photoconverted cells (closed symbols) is a consequence of synthesis and maturation of fresh eGFP with longer lifetime which gradually elevates the measured lifetime. As apparent from Figure 6C,D, this rise of about 50 ps in 80 min is almost insignificant. All G_NPMmut is relocalized to the nucleus at that time.

**Figure 7:**
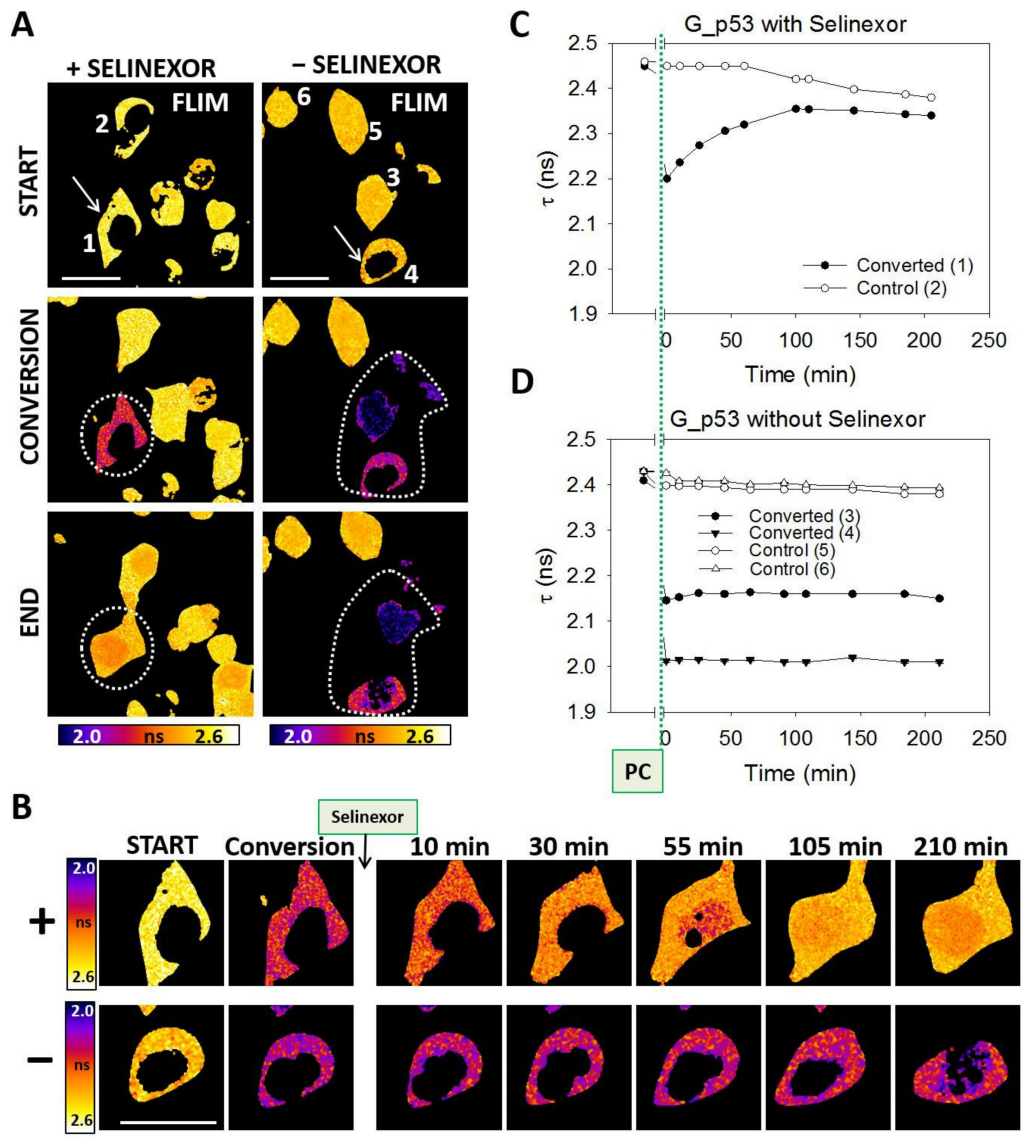
Effect of Selinexor on localization and lifetime of G_p53 co-expressed with R_NPMmut in HEK-293T cells. (**A**) Lifetime images of G_p53 in the presence and absence of 4 µM Selinexor before (the 1st row), immediately after (the 2nd row) and 210 min after the photoconversion (PC) (the 3rd row). The photoconverted area is bordered by the dashed line. Bar represents 20 µm; (**B**) detailed time evolution of G_p53 localization and τ_GFP_ maps in single cell with photoconverted eGFP in the presence (upper row, cell no. 1) and absence of Selinexor (lower row, cell no. 4). The cells are marked by arrows in panel A. The nuclear signal was negligible in the absence of Selinexor. Bar is 20 µm; (**C**,**D**) time evolution of τ_GFP_ in the photoconverted (1, 3, 4) and control (2, 5, 6) cells in the presence (**C**) and absence (**D**) of Selinexor. Cells used for integration of the plotted signal are marked in the panel A. List of abbreviations is in Table 1. Representative results from 3–5 repeated experiments are shown.

Then we performed similar relocalization experiments with cells expressing G_p53 accompanied by R_NPMmut to ensure cytoplasmic localization of G_p53, Figure 7. We selected cells with marked amount of G_p53 in the cytoplasm, photoconverted those cells and mapped evolution of τ_GFP_. Figure 7A depicts FLIM images of the photoconverted and control cells in the presence and absence of Selinexor. The process is shown again in more detail in Figure 7B (cells no. 1 and 4 from panel A, marked by arrows). Compared to G_NPMmut examined in Figure 6, the G_p53 nuclear relocation is slower and incomplete within 3 h. The relatively slow return of G_p53 to the nucleus was accompanied by a faster, significant increase of τ_GFP_ upon the application of Selinexor. This is best seen in panels C and D where evolution of τ_GFP_ averaged over individual cells is shown. We can see that while control cells without Selinexor exhibit almost no lifetime change, Figure 7D, the addition of the drug induces significant increase of τ_GFP_ in the photoconverted cell (cell no. 1, panel A), Figure 7C. Since the eGFP photoconversion was shown to be irreversible and fluorescence lifetimes stable in time [55], the observed increase in τ_GFP_ should be caused either by de novo synthesis of fresh long-lifetime G_p53 or by degradation of the short-lifetime photoconverted protein. The process reaches its plateau at about 2 h with the characteristic rise half-time (T_1/2_) of about 60 min. Some decrease in τ_GFP_ of the control unconverted cell in the presence of Selinexor (cell no. 2, panel A) is likely caused by a gradual reimport of eGFP-tagged p53 to a dense nucleus, where eGFP exhibits slightly lower lifetime clearly visible at the end of the experiment, Figure 7A. Such eGFP lifetime shortening in a dense environment modulating the refraction index has been commonly observed [55,65].

**Figure 8:**
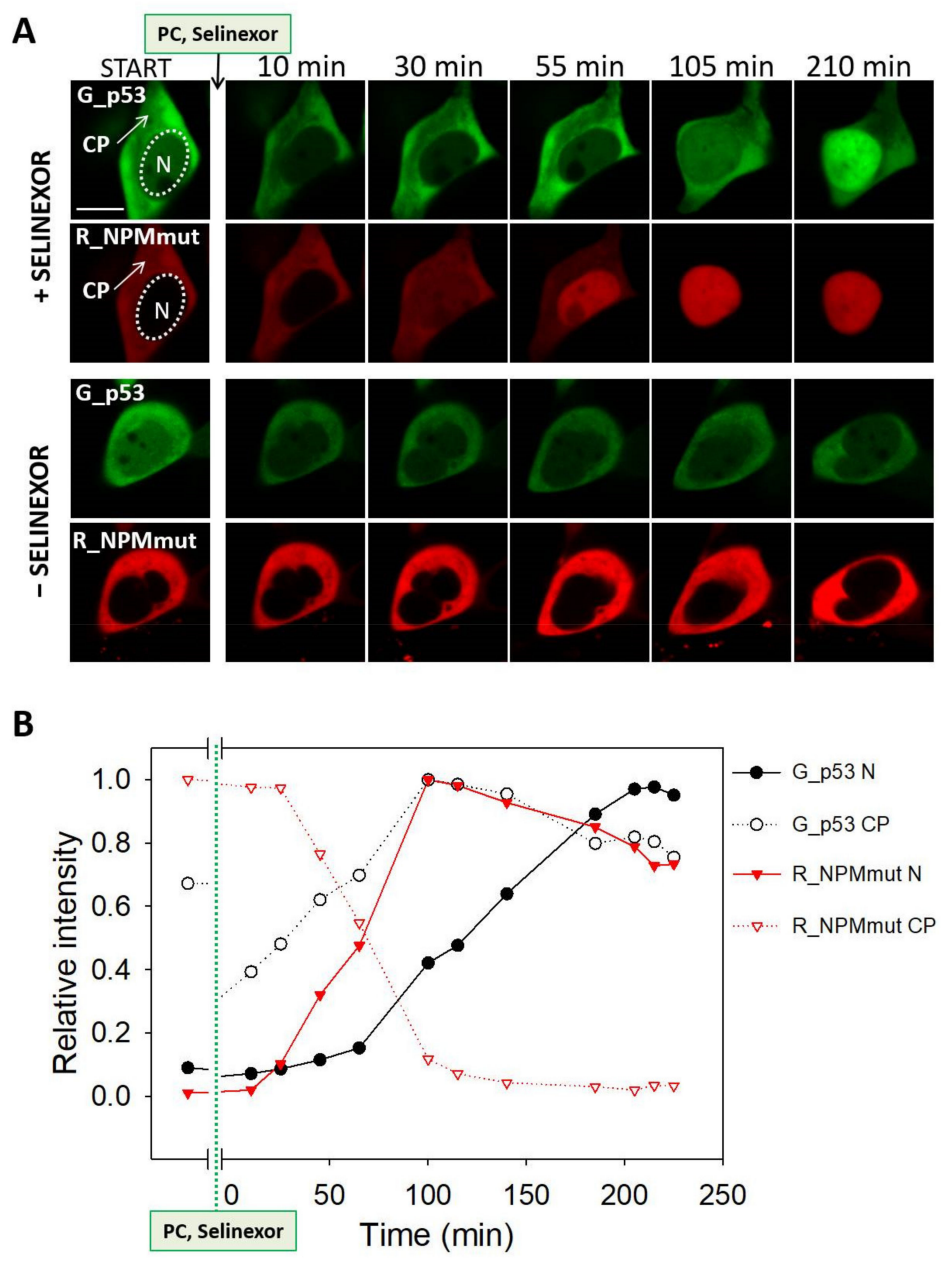
Effect of Selinexor on the fluorescence intensity and localization of G_p53 and R_NPMmut co-transfected in HEK-293T cells. (**A**) Intensity images of G_p53 co-expressed with R_NPMmut at different times upon the addition of 4 µM Selinexor (upper two rows, cell no. 1). The control experiment without Selinexor is presented in the lower two rows (cell no. 4). Depicted are cells from Figure 7B. Bar represents 10 µm; (**B**) fluorescence intensity changes of G_p53 (circles) and R_NPMmut (triangles) in the nucleus (close symbols, full lines, N) and the cytoplasm (open symbols, dash lines, CP) of the cell from the upper two rows of the panel A. Areas used for integration of the fluorescence signal are separated by the dotted line in panel A. The initial irreversible photoconversion (PC) of G_p53 reduced eGFP intensity to about 45% of its initial value before the addition of Selinexor (open circles). List of abbreviations is in Table 1. Representative results from 3–5 repeated experiments are shown.

Selinexor-induced synthesis/maturation of fresh G_p53 supposedly causing the observed increase in τ_GFP_ should be accompanied by an increase in the emission intensity. Indeed, increasing fluorescence of exogenous G_p53 was observed for both converted and unconverted cells upon the Selinexor addition, as documented in Figure 8 and Figure S4, respectively. In Figure 8, relocalization dynamics of G_p53 and R_NPMmut in photoconverted cells from Figure 7 (cells no. 1 and 4) is shown in detail. Upper rows of Figure 8A display a gradual relocalization of G_p53 and R_NPMmut after the addition of Selinexor. It can be seen that while both proteins gradually relocalize to the nucleus, relocalization of R_NPMmut appears faster. Lower two rows containing control data without Selinexor do not indicate any evolution of the protein amount and distribution. Time evolution of fluorescence signal in the nucleus and cytoplasm of the Selinexor-treated cell from Figure 8A is shown in Figure 8B. From the intensity increase in the nucleus we can conclude that NPMmut re-localizes to the nucleus about twice faster than p53, half-rise times T_1/2_ being of about 60 and 120 min for NPMmut and p53, respectively. Such asynchronous behavior indicates, that NPMmut and p53 may respond independently to the nuclear export inhibition. Interestingly, despite outflux of p53 from the cytoplasm, photoconversion-reduced cytoplasmic intensity of G_p53 rises in a response to Selinexor. The observation is fully consistent with the increased τ_GFP_ documented in Figure 7C. Kinetic behavior is also rather similar being completed in about 2 h. Similar rise in the fluorescence intensity was observed also for unconverted cells. Figure S5 demonstrates that the cytoplasmic emission quickly rises from the non-reduced pre-treatment level and is followed by a decrease caused by p53 outflux to the nucleus, Figure S5B (open circles). Presented results strongly support conclusion that Selinexor upregulates de novo synthesis of G_p53 in live cells. In agreement with another drug from the SINE family, KPT-185 [66], we can attribute the observed p53 up-regulation to the Selinexor-induced stabilization and activation of p53 [67].

**Figure 9:**
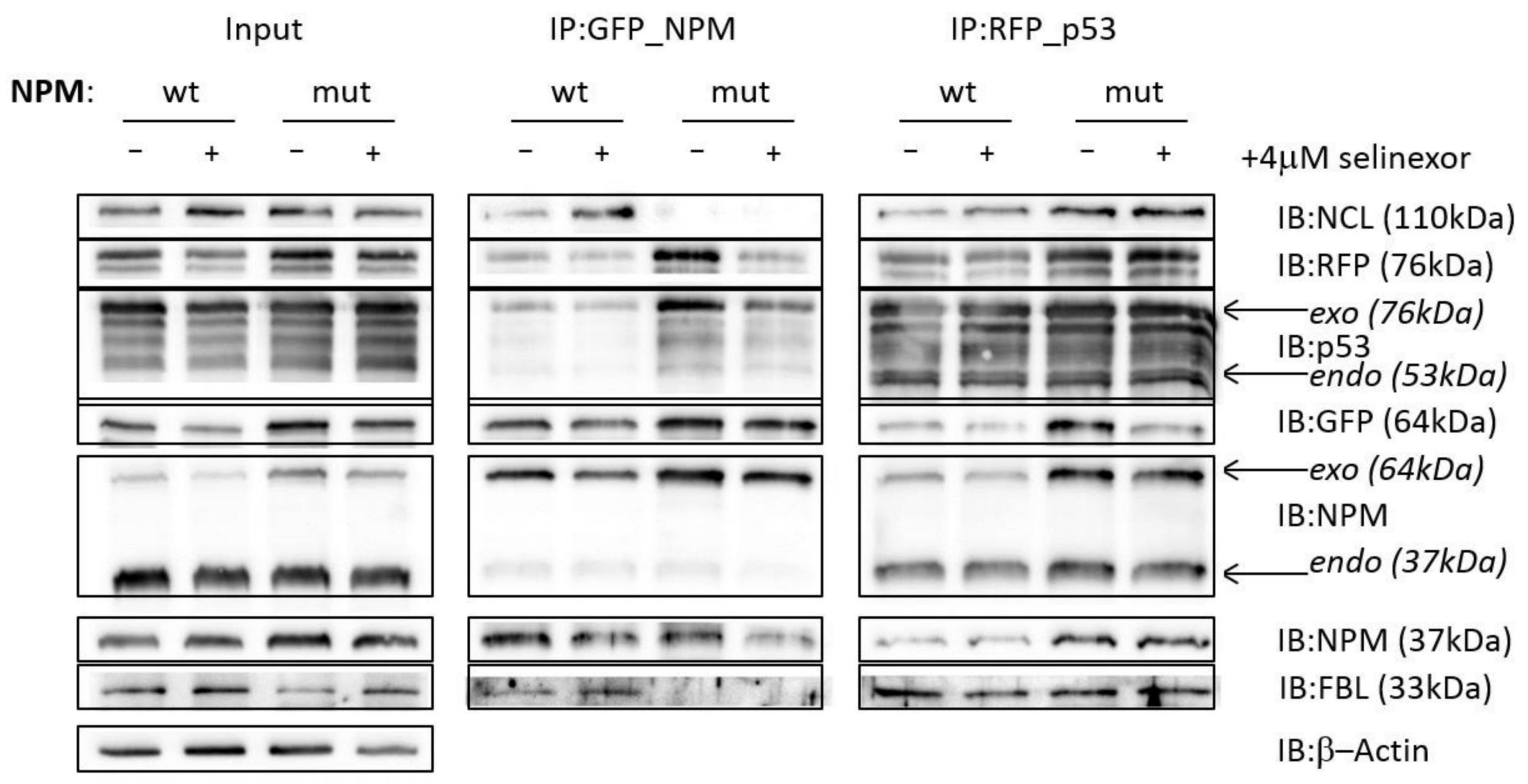
Effect of Selinexor on interaction of p53 with NPM variants in lysates of HEK-293T cells. The cells were co-transfected with G_NPM variants (wt, mut) and R_p53 in duplicates. Selinexor (4 µM) was added to one sample of each variant 2 h before the GFP/RFP-precipitation. Levels of co-precipitated proteins were investigated by immunoblotting. β-Actin expression served as a loading control. List of abbreviations is in Table 1.

Effect of Selinexor on the p53-NPMwt/mut interaction was further tested by immunoprecipitation. We performed GFP/RFP immunoprecipitation from Selinexor-treated cells co-transfected with G_NPMwt or G_NPMmut, and with R_p53, Figure 9. We found comparable amounts of the p53/NPM complexes in all samples irrespectively of the presence of Selinexor. The result suggests that the complex forms independently of the Selinexor treatment. Selinexor had no effect on NPMwt interaction with NCL as well, which was used as a control for immunoprecipitation. In accordance with our previous findings [28], NCL was not detected in NPMmut precipitates. Moreover, NCL was found in all p53 precipitates confirming interaction of these two proteins [5]. Interestingly, identical results as for NCL were obtained also for another nucleolar protein, fibrillarin (FBL). Specifically, FBL interaction with NPMwt was inhibited by the NPM mutation and FBL interacted with p53, Figure 9. These findings are to the best of our knowledge novel.

**Figure 10:**
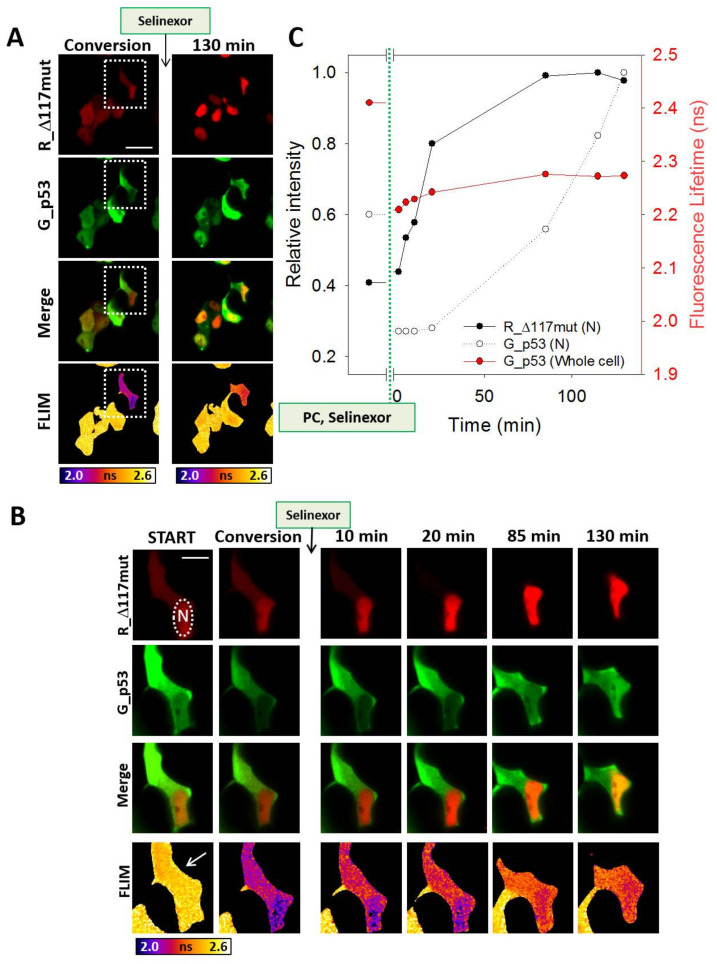
Cellular dynamics of G_p53 and R_Δ117mut upon Selinexor treatment. (**A**) Overview of localization, intensity and lifetime changes of co-expressed G_p53 and R_Δ117mut in HEK-293T cells after addition of 4 µM Selinexor. eGFP within the region bordered by the dashed line was irreversibly photoconverted (PC) to the low-lifetime form before the Selinexor addition. Bar represents 30 µm; (**B**) detailed time evolution of the photoconverted region from panel A. Nucleus (N) is marked by the dashed line. Bar represents 10 µm; (**C**) intensity change of G_p53 (open circles) and R_Δ117mut (closed circles) at the nucleus of the cell from Panel B. Signal for the lifetime change was integrated from the whole cellular area (red circles). List of abbreviations is in Table 1. Representative results from 3–5 repeated experiments are shown.

The asynchronous reimport of the complex components back to the nucleus was observed in transfected HEK-293T cells also when p53 was translocated to the cytoplasm by Δ117mut, see Figure 10. The panel A shows, from the top to bottom, intensity images of G_p53, R_Δ117mut, their merge, and FLIM images just after the PC and 130 min later. Relocalization dynamics in the photoconverted region is shown in detail in Figure 10B. Rapid relocation of R_Δ117mut in the nucleus, gradual increase of G_p53 nuclear intensity as well as slight increase of τ_GFP_ in the photoconverted area can be seen upon the addition of Selinexor. Based on the nuclear rise in the R_Δ117mut emission intensity depicted in Figure 10C, it seems that Δ117mut relocalizes back to the nucleus even faster than NPMmut shown in Figure 7, with T_1/2_ of about 20–30 min. Nevertheless, it should be noted that the relative amount of Δ117mut in the cytoplasm was lower than for NPMmut prior the Selinexor addition, compare Figure 8A and Figure 10B. It is also evident that similarly to NPMmut, the double mutant Δ117mut re-localizes to the nucleus much faster than p53. Increase in τ_GFP_ of G_p53 integrated across the whole cell does not exhibit so marked elevation in presence of Δ117mut, suggesting lower Selinexor-induced upregulation compared to cells with NPMmut. Mechanistic reasons for this difference are unknown at the moment.

### 3.5. Interaction of p53 and NPM in AML Cell Lines

**Figure 11:**
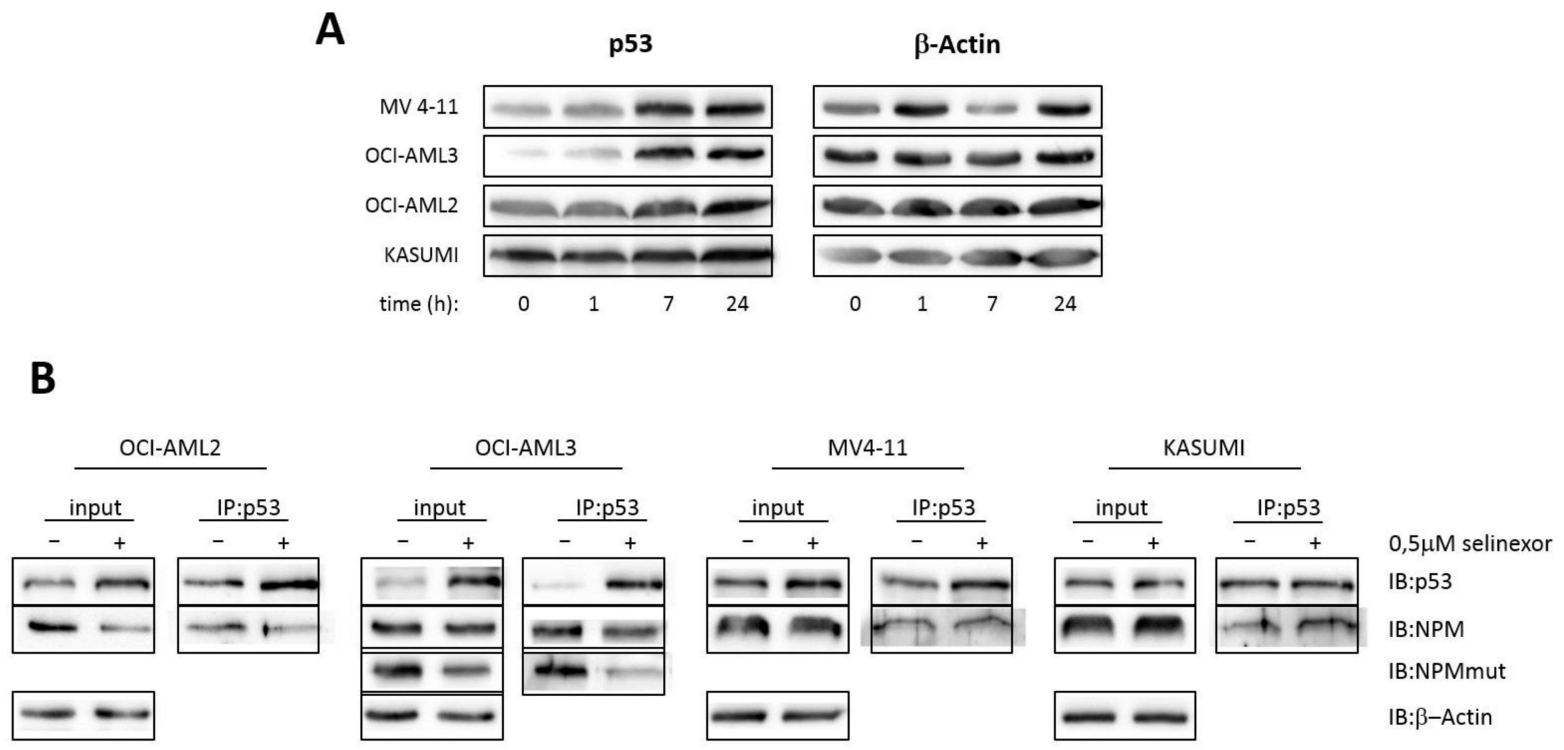
Effect of 0.5 µM Selinexor on interaction between p53 and NPM in AML-derived cell lines. (**A**) p53 expression at indicated times after the Selinexor addition; (**B**) p53-precipitation 24 h after the Selinexor addition. Control samples without Selinexor were collected at the same time. Levels of co-precipitated NPM were investigated by immunoblotting. β-Actin expression served as a loading control.

The HEK-293T cell line is suitable for simulations with fluorescently tagged proteins thanks to its highly effective transfectability. Nevertheless, its p53 signaling may be different from that in leukemia cells. We therefore performed immunoprecipitation experiments on a set of leukemia cell lines, Figure 11. Our choice of the cell lines was representative of AML-characteristic mutations. OCI-AML2 cells carry DNMT3A (R635W) mutation, OCI-AML3 line contains NPM1 and DNMT3A (R882C) mutations, and MV4-11 cells have FLT3-ITD mutation. All these lines express wild type p53. The fourth tested line, KASUMI-1, carries the p53 mutation (R248Q) causing high p53 expression in intact cells. We detected Selinexor-induced increase of p53 levels in cell lysates of all cell lines expressing p53wt, Figure 11A. Despite the rise in p53 even in cell lines lacking NPMmut (i.e., expressing only NPMwt), the p53 expression increases faster in OCI-AML3 containing NPM with the mutated C-terminus. Levels of p53-dependent proapototic proteins p21 and Puma were also elevated, specifically in OCI-AML3. Concurrently, antiapoptotic protein Survivin, which is also regulated by p53, was lowered in Selinexor-treated AML cell lines (Figure S6). To confirm persisting NPM-p53 interaction observed in Selinexor-treated cells transfected with the exogenous FP-labeled proteins, we performed similar experiments with our panel of AML-related cell lines, Figure 11B. Due to the higher sensitivity of AML cells to the treatment, the concentration of Selinexor was lowered eight-fold to 0.5 µM and the exposure of cells to the drug was prolonged from 2 h to 24 h. In agreement with the results on HEK-293T cells, NPM co-precipitated with p53 in all examined cell lines irrespectively of the Selinexor presence. In the OCI-AML3 cell line, NPMmut was also detected with a mutant-specific antibody in the p53 precipitates.

## 4. Discussion

A presence of interaction between NPM and p53 was reported in stressed cells almost twenty years ago [3]. Since then, the interaction between purified recombinant proteins was confirmed by surface plasmon resonance and regions responsible for the interaction were specified in GST-pulldown assays [4]. As the C-terminus of NPM (AA242-269) mediates the interaction with p53 [4], the interaction is expected to be disrupted by the AML-associated mutation located at the same terminus. However, no data confirming this hypothesis have been published yet. In this work we bring several lines of evidence that both p53-NPMwt and p53-NPMmut interactions occur under physiological conditions in live cells.

We have reported consistently with others that due to the heterooligomer formation with NPMmut, a fraction of NPMwt is delocalized into the cytoplasm [34,35]. Similarly, confocal microscopy revealed extensive p53 delocalization into the cytoplasm of cells co-expressing NPMmut, Figure 1, which suggests interaction between p53 and NPMmut. The complex formation was therefore investigated by co-immunoprecipitation using GFP Trap beads. The experiments have confirmed the published p53-NPMwt interaction [3,4]. We newly proved interaction between p53 and C-terminally mutated variants of NPM that are characteristic for AML, Figure 2. Our discovery of the p53-NPMmut interaction appears rather surprising in the light that NCL, which also interacts with C-terminal NPMwt region (AA187-241) [21], was not detected in the complex with NPMmut [28]. Interaction of p53 with both NPMwt and NPMmut was further confirmed in live cells, using FLIM-FRET, Figure 5. Subsequently, p53 was found also in the G_Δ117wt-precipitates, which suggests that the NPM oligomerization domain is unessential for its binding to p53, see Figure 4A. In fact, even higher amount of p53 co-precipitated with Δ117wt than with NPMwt. FRET between G_Δ117wt and R_p53 confirmed this finding in live HEK-293T cells, Figure 5A. We conclude that neither the wild-type C-terminus nor the oligomeric NPM form is necessary for p53 binding.

We therefore tested how the double mutation affects formation of p53/NPM complex. Massive cytoplasmic localization of fluorescently labeled p53 was observed when co-expressed with Δ117mut in live HEK-293T cells, as shown in Figure 3. Surprisingly, the cells abundantly exhibited p53 in the cytoplasm, despite the prevailingly nuclear localization of Δ117mut. The result was the same for both eGFP and mRFP1 labeling combination, see Figure S4. This observation seems not to be completely in line with the finding that lower p53-Δ117mut binding was detected by co-immunoprecipitation, Figure 4. This suggests that the p53/Δ117mut complex is formed, nevertheless its k_D_ is likely higher and lower amount of the complex is therefore detected by immunoprecipitation. This conclusion is supported also by FLIM results in Figure 5B where only insignificant FRET stemming from a small fraction of the R_p53/G_Δ117mut complex in the cytoplasm was detected on a high background of free uncomplexed G_Δ117mut donor. Due to the very low amount of R_p53 in the nucleus, the complex cannot be detected there by FLIM-FRET, either.

Both p53 and NPM interact with nuclear exporter XPO1. However, only in cells expressing NPMmut, p53 is ubiquitously detected in the cytoplasm. As NPM forms complex with p53 [3,4] and we found this complex in intact cells, we expect that p53 and NPMmut are simultaneously co-exported out of the nucleus in the p53/NPMmut complex. Our data suggest that even weaker binding of p53 to Δ117mut facilitates translocation of p53 to the cytoplasm. However, an alternative transport mechanism involving ternary complex with some other NPM- and p53-interacting partner, cannot be ruled out at this moment.

The translocation of p53 to the cytoplasm implicates important consequences for its turnover and function. In intact cells, p53 expression is tightly controlled by numerous posttranslational modifications and it is kept at low level by proteasome. However, stable p53 production from *TP53* transcript allows for rapid reaction on stress stimuli by stopping the protein degradation and triggering its transcription activity. The regulation of the p53 expression, stability and activity takes place mainly in the nucleus and the p53 delocalization likely causes a loss of its function [39,68]. Targeting the cytoplasmic p53 and restoration of its nuclear localization could be therefore a promising alternative for the therapy of the AML with NPMmut. An inhibition of XPO1 by Selinexor [43,69] or any other clinically efficient inhibitor, e.g., verdinexor [70], seems to be an auspicious choice [41].

When the XPO1 exporter is blocked, NPMmut is no longer exported to the cytoplasm and nuclear export/import equilibrium is shifted toward the relocalization of NPMmut into the nucleus [51]. In co-transfected cells treated with Selinexor, NPMmut thus cannot drive p53 translocation to the cytoplasm in the p53/NPMmut complex. At the same time, p53, as another XPO1 client, cannot be exported to the cytoplasm and returns to the nucleus via importins [71]. Indeed, we observed relocalization of both NPMmut and p53 to the nucleus upon addition of 4 µM Selinexor to transfected HEK-293T cells, Figure 6 and Figure 7. The observed Selinexor-induced increase of p53 expression is in accordance with the activation of p53-dependent apoptosis after the Selinexor treatment [67,69]. Importantly, p53 and NPMmut responded independently and exhibited different dynamics upon the Selinexor addition. Figure 8 and Figure S4 demonstrate that the redistribution of NPMmut after application of Selinexor is considerably faster than that of p53. It seems evident that p53 and NPMmut do not have to be synchronously co-transported to the nucleus. Our new results indicate that cytoplasmic complexes of p53 with C-terminally-mutated NPM variants are weaker and rather dynamic. Exchanging complex constituents and rapid equilibration could allow for independent nuclear import of p53 and NPMmut variants. Abolishment of the p53-NPM interaction by Selinexor is therefore not required. The detection of the p53/NPMmut complex in Figure 9 is fully consistent with the outlined mechanism.

Cytoplasmic mislocalization of p53 requires presence of NPMmut. Targeting of the NPMmut-p53 interaction might therefore facilitate restoration of the p53 proapoptotic and regulative function in the nucleus. Nevertheless, strong translocation of p53 to the cytoplasm was observed in the presence of Δ117mut when lower amount of the p53-NPM complex is expected to be formed in the nucleus independently of the Selinexor, owing to the documented weaker p53-Δ117mut interaction. The faster reimport of Δ117mut to the nucleus, compared to that of NPMmut where the interaction is stronger, is therefore consistent with a higher cytoplasmic pool of free Δ117mut being ready for import. The observed asynchronous reimport of p53 and NPMmut suggests that an induction of the p53 nuclear translocation rather than a disruption of the p53-NPMmut interaction could stabilize p53 and restore its cellular function. Therapeutic boosting of the p53 reimport could be the right target, since p53 is also a subject of ubiquitinylation and fast degradation in the cytoplasm [42].

The therapeutic effect of Selinexor is supposed to be mediated by restoration of the nuclear localization of p53. This could happen to some extent in all cells; however, the effect should be augmented in cells with mislocalized p53. Significant induction of p53 is visible in Figure 11A and Figure S7 for the OCI-AML3 cell line expressing NPMmut. Interestingly, NPMmut moves to the nuclei during the first hours of the Selinexor treatment and in 24 h localizes mostly in the nucleoli (Figure S7). Intact OCI-AML3 cells display very low basal levels of p53, likely caused by its high degradation when in the cytoplasm. Fast p53 induction in the presence of Selinexor then could be partially explained by its restored localization and a lower degradation rate there. However, due to the very low p53 concentration in intact cells, the supposed NPMmut-dependent translocation of p53 to the cytoplasm of OCI-AML3 cells cannot be checked by immunostaining (Figure S7). The increase in p53 was accompanied by changes in p53-dependent apoptosis-related proteins, specifically by enhanced expression of p21 and Puma and decreased antiapoptotic protein Survivin. Survivin was shown to be upregulated in AML cells and further enhanced by a standard cytarabine treatment [72]. Selinexor might therefore overcome the cytarabine resistance by Survivin inhibition. Slight PARP fragmentation and caspase-3 activation at 24 h upon the treatment indicates further evolution of the apoptotic process (Figure S6). Selinexor-induced phosphorylation on several Serine residues responsible for p53 activation was also detected (Figure S6). Amplified p53 transcriptional activity in response to UVC radiation has been recently reported in cells with aberrantly phosphorylated NPM [73]. In their work, silencing of dual-specificity phosphatase 3 (DUSP3) led to NPM dephosphorylation on tyrosines Y29, Y67 and Y217. Increased NPM monomer/oligomer ratio and enhanced p53-S15 phosphorylation has been observed as well [73]. Strong induction of p53 in Figure 11B accompanied by signs of lowered NPMmut level in the Selinexor-treated OCI-AML3 suggests that such large p53 increase could be related to the presence of NPMmut. Despite the NPMmut decrease in Figure 11B was not statistically significant (*N* = 3), we have previously shown that NPM mutation enhances the NPM monomer/oligomer ratio [28]. Therefore, it raises a question whether the NPM mutation could alter NPM phosphorylation with expected effects on p53 activation. We conclude that induction of p53 might explain the positive therapeutic effect of Selinexor in the treatment of AML with NPMmut, possibly in combination with other p53-inducing agents, such as mdm2/p53 inhibitors, e.g., nutlin-3a [43].

## 5. Conclusions

We examined importance of p53-NPM interaction for accumulation of p53 in the cytoplasm. The interaction was found to be unaffected by the AML-related mutation of the NPM C-terminus. We newly proved that the p53-NPMmut interaction persists also when the oligomerization of NPM is compromised by the deletion of its N-terminal domain (AA1-117). The cytoplasmic localization of exogenous p53 was found also in the presence of NPM double mutant Δ117mut co-expressed in live HEK-293T cells. To the best of our knowledge, the NPMmut-mediated cytoplasmic translocation of p53 has not been reported yet.

We documented that in response to the Selinexor treatment, both p53 and NPMmut returned to the nucleus, however, with significantly different kinetics. We concluded that p53 and NPMmut do not have to be co-transported to the nucleus as stable complexes. They rather form weaker short-lived complexes allowing rapid exchange of its constituents and independent nuclear import. This new insight opens alternative ways and targets for the AML therapy.

## Figures and Tables

**Table 1:** List of constructs. All protein constructs except PAK2_eGFP are tagged on their N-terminus. Symbol “/” denotes mixture of constructs in text of the article.

Construct	Abbreviation	Construct	Abbreviation	Construct	Abbreviation
eGFP_NPMwt	G_NPMwt	eGFP_Δ117NPMwt	G_Δ117wt	eGFP_p53wt	G_p53
mRFP1_NPMwt	R_NPMwt	mRFP1_Δ117NPMwt	R_Δ117wt	mRFP1_p53wt	R_p53
eGFP_NPMmutA ^1^	G_NPMmut	eGFP_Δ117NPMmutA	G_Δ117mut	PAK2_eGFP ^2^	G_PAK2
mRFP1_NPMmutA	R_NPMmut	mRFP1_Δ117NPMmutA	R_Δ117mut		

^1^ See [36] for the description of the NPM mutant type A (NPMmutA). ^2^ See [54] for the description of plasmid for expression of PAK2_eGFP.

**Table 2:** Summary of interactions and localizations of p53 and the respective NPM variant specified in the first column. The left section outlines interactions of p53 with NPM variants and compares the immunoprecipitation and FLIM-FRET results. The right section schematically summarizes cellular localization of the respective fusion proteins. Abbreviations are defined in Table 1.

	*Interaction of p53 and NPM Variants*		*Localization of the Fusion Proteins*			
***Co-expressed fusion proteins***	*Immunoprecipitation results*	*FRET results*	*eGFP-tagged p53*	*Cytoplasmic signal*	*mRFP1-tagged NPM variant*	*Cytoplasmic signal*
***G*_*p53* /** ***R*_*NPM*** ***wt***	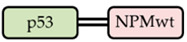	✓	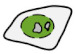	−	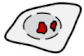	−
***G*_*p53* /** ***R*_*NPM*** ***mut***	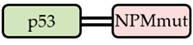	✓	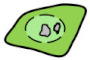	+	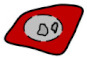	+ +
***G*_*p53* /** ***R*_ ** **Δ *117wt***	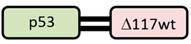	✓	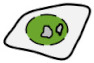	−	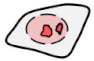	−
***G*_*p53* /** ***R*_ ** **Δ *117mut***	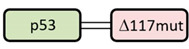	X	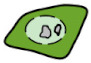	+ +	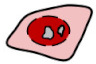	+

## Data Availability

The data presented in this study are available in Supplementary Materials, see above.

## Supplementary Materials

The following are available online at https://www.mdpi.com/article/10.3390/cancers13133266/s1, Figure S1: Localization of distinct fluorescently labeled proteins expressed in HEK-293T cells with nuclei counterstained with Hoechst33342. Figure S2: Localization of p53 co-expressed with NPMmut in HEK-293T cells shown for two tagging combinations; Figure S3: Interaction between NPMwt and p53 in cell lysates of transfected HEK-293T cells; Figure S4: Localization of p53 co-expressed with Δ117mut in HEK-293T cells shown for two tagging combinations; Figure S5: Effect of Selinexor on the fluorescence intensity and localization of G_p53 and R_NPMmut co-transfected in HEK-293T cells. Figure S6: Expression of phosphorylated p53 variants and apoptosis-related proteins in AML cell lines after treatment with 0.5µM Selinexor. Figure S7: Localization of p53 stained with Alexa Fluor488 (green) and total NPM stained with Alexa Fluor555 (red) in OCI-AML3 cells treated with 0.5 µM Selinexor. Table S1: Specification of primary antibodies used for protein detection in immunoblotting. Table S2: Specification of primary antibodies used for immunoblotting of phosphorylated p53 and apoptosis-related proteins (Figure S7). Supplementary Material 2 contains original Western Blot images.
