# Supplementary material for: AML-Related NPM Mutations Drive p53 Delocalization into the Cytoplasm with Possible Impact on p53-Dependent Stress Response
Source: Cancers (Basel). 2021 Jun 29;13(13):3266. doi: 10.3390/cancers13133266 (PMC8269334; doi:10.3390/cancers13133266)

Supplementary material 2

original whole blots with MW markers for Western blot results

(numbers represent relative protein levels as evaluated by densitometry for each band)

AML-related NPM mutations drive p53 delocalization into the cytoplasm with possible impact on p53-dependent stress response

Holoubek A[1]., Strachotová D.[2], Otevřelová P.[1], Röselová P.[1], Heřman P.[2], Brodská B[1].

[1]Department of Proteomics, Institute of Hematology and Blood Transfusion, U Nemocnice 1, 128 20 Prague, Czech Republic

[2]Faculty of Mathematics and Physics, Institute of Physics, Charles University, Ke Karlovu 5, 121 16 Prague 2, Czech Republic

MWs of marker (Pink protein ladder, CE Biosystems, Czech Republic) in 12,5% SDS-PAGE (Tris-Glycine):

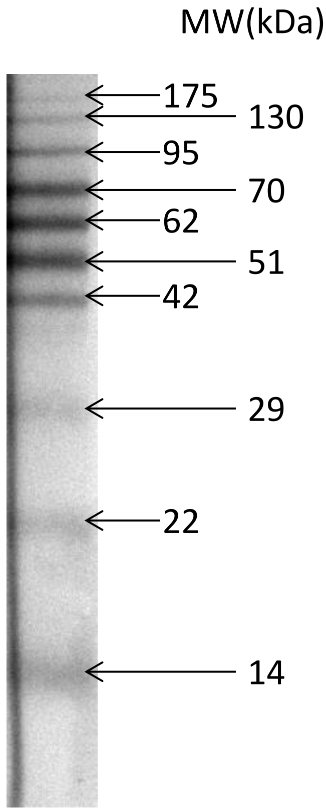

Fig.2A

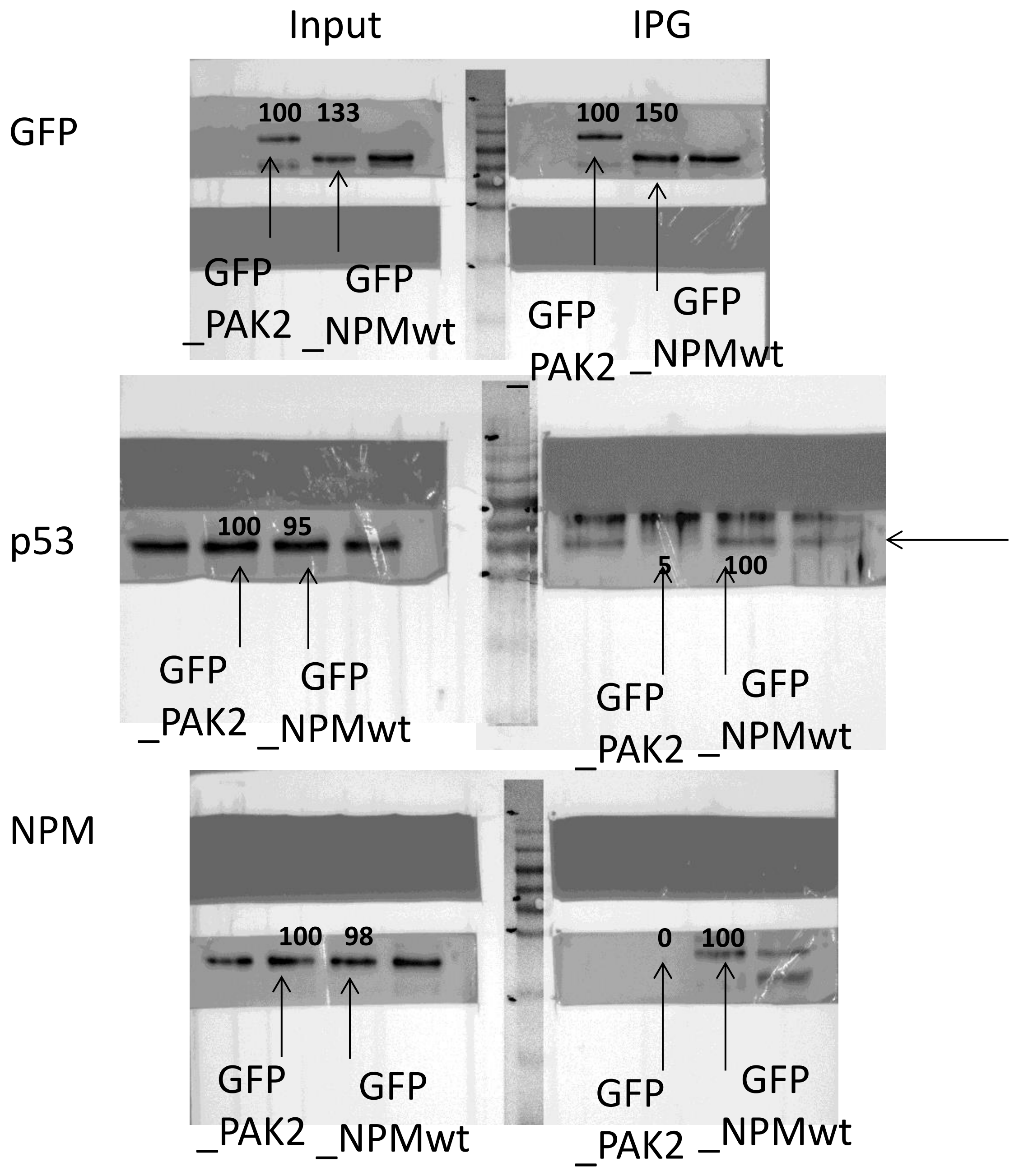

Input
IPG
GFP
100 133
100 150
GFP _PAK2
GFP _NPMwt
GFP _PAK2
GFP _NPMwt
p53
100 95
5 100
GFP _PAK2
GFP _NPMwt
GFP _PAK2
GFP _NPMwt
NPM
100 98
0 100
GFP _PAK2
GFP _NPMwt
GFP _PAK2
GFP _NPMwt

Fig.2B

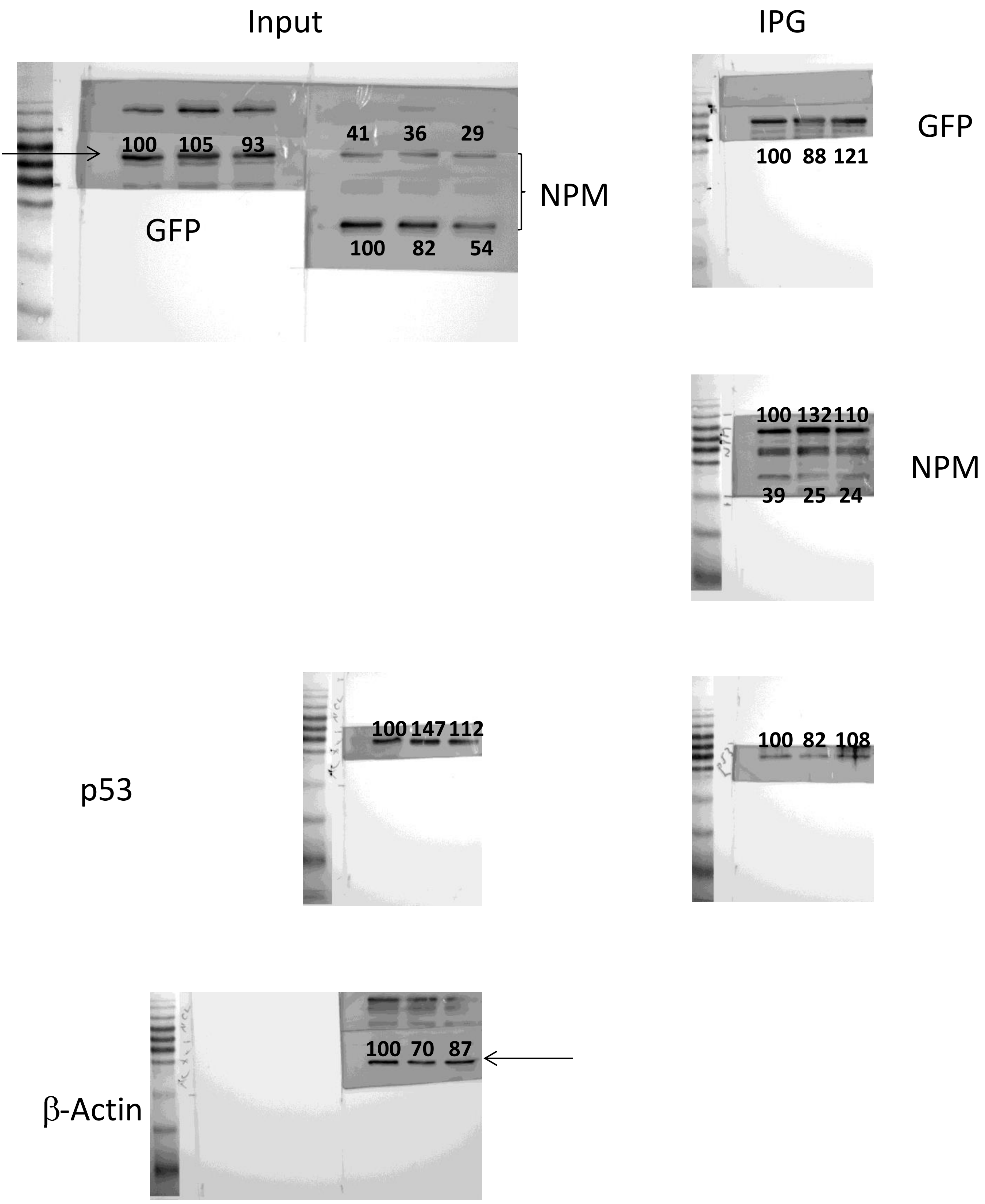

Input
IPG
100 105 93
41 36 29
GFP
NPM
100 82 54
GFP
100 88 121
100 132 110
39 25 24
NPM
p53
100 147 112
100 82 108
β-Actin
100 70 87

Fig.4A

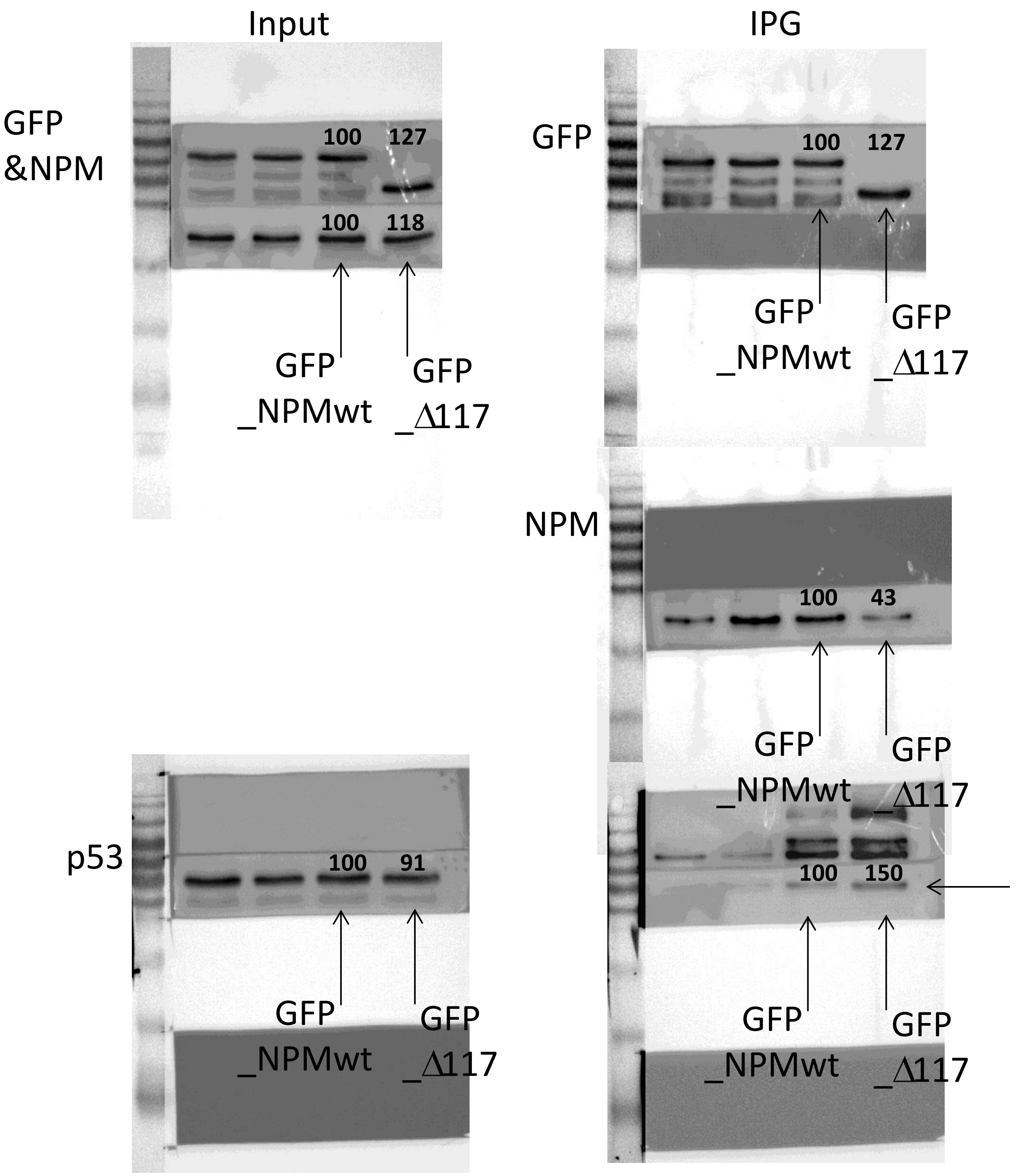
Input
IPG
GFP
&NPM
100
127
100
118
GFP
_NPMwt
GFP
_Δ117
GFP
100
127
GFP
_NPMwt
GFP
_Δ117
NPM
100
43
GFP
_NPMwt
GFP
_Δ117
p53
100
91
GFP
_NPMwt
GFP
_Δ117
100
150
GFP
_NPMwt
GFP
_Δ117

Fig.4B

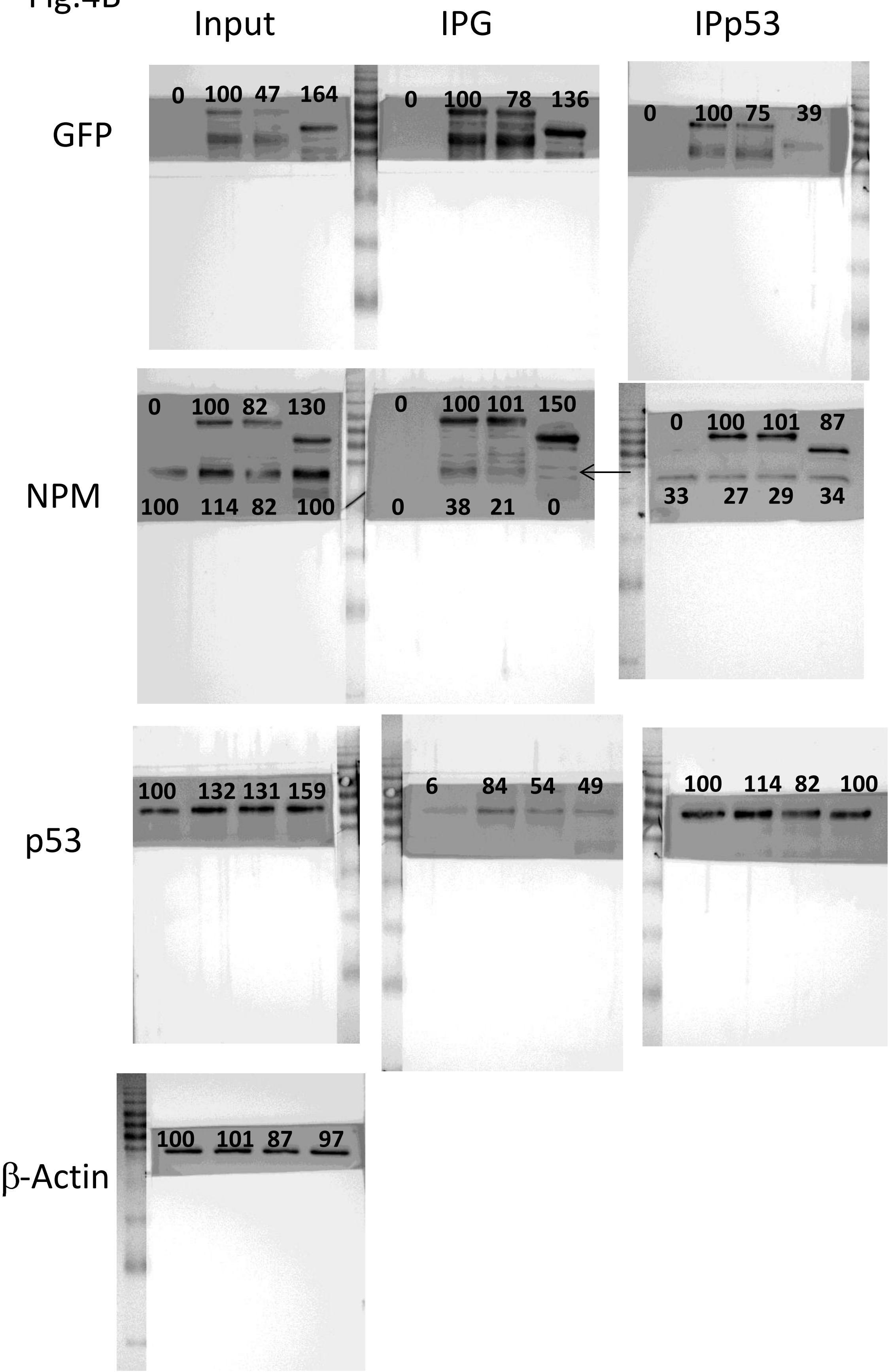
Input
IPG
IPp53
GFP
0 100 47 164
0 100 78 136
0 100 75 39
NPM
0 100 82 130
100 114 82 100
0 100 101 150
0 38 21 0
0 100 101 87
33 27 29 34
p53
100 132 131 159
6 84 54 49
100 114 82 100
β-Actin
100 101 87 97

Fig.9

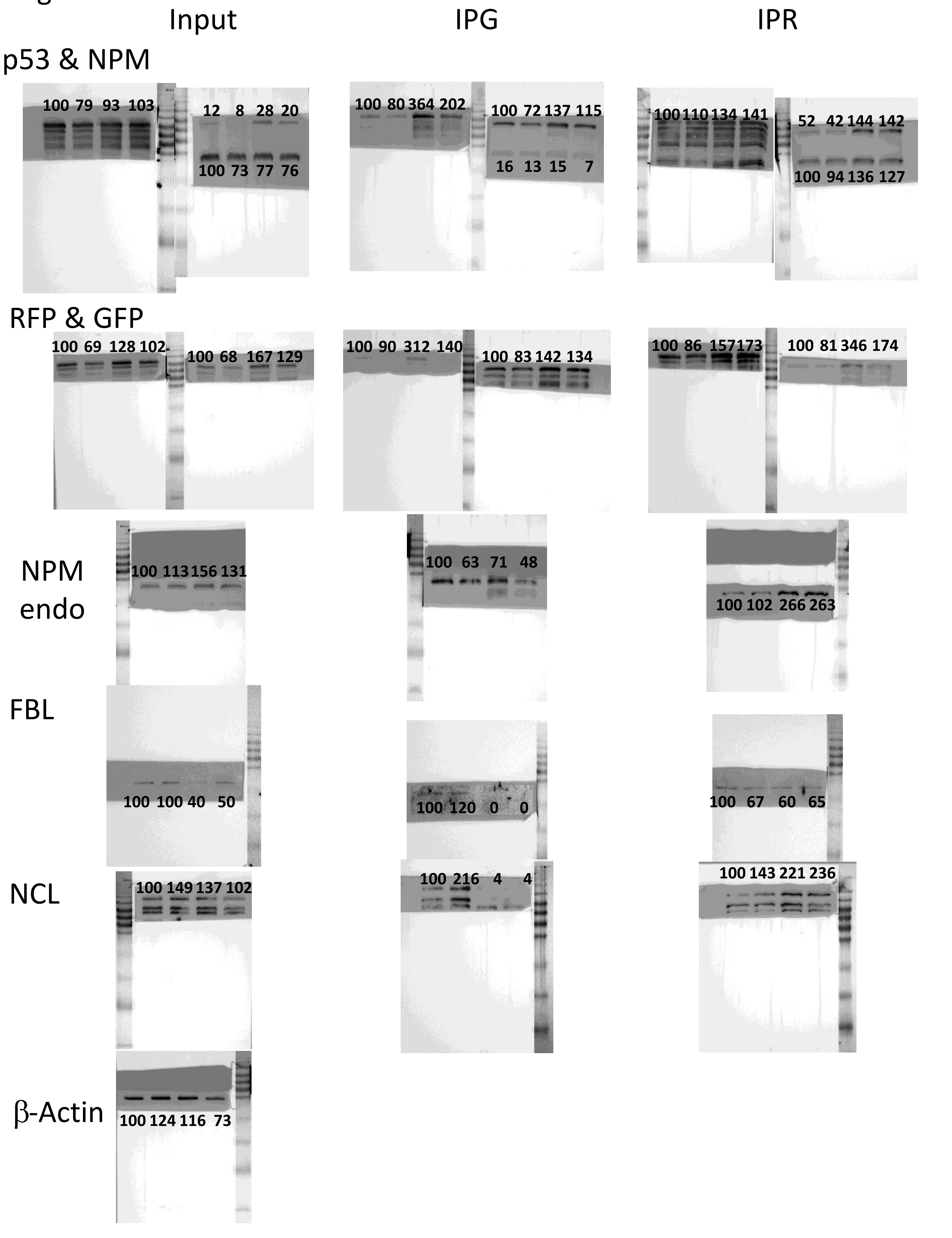
Input
IPG
IPR
p53 & NPM
100 79 93 103
12 8 28 20
100 73 77 76
100 80 364 202
100 72 137 115
16 13 15 7
100 110 134 141
52 42 144 142
100 94 136 127
RFP & GFP
100 69 128 102
100 68 167 129
100 90 312 140
100 83 142 134
100 86 157 173
100 81 346 174
NPM endo
100 113 156 131
100 63 71 48
100 102 266 263
FBL
100 100 40 50
100 120 0 0
100 67 60 65
NCL
100 149 137 102
100 216 4 4
100 143 221 236
β-Actin
100 124 116 73

Fig.11A

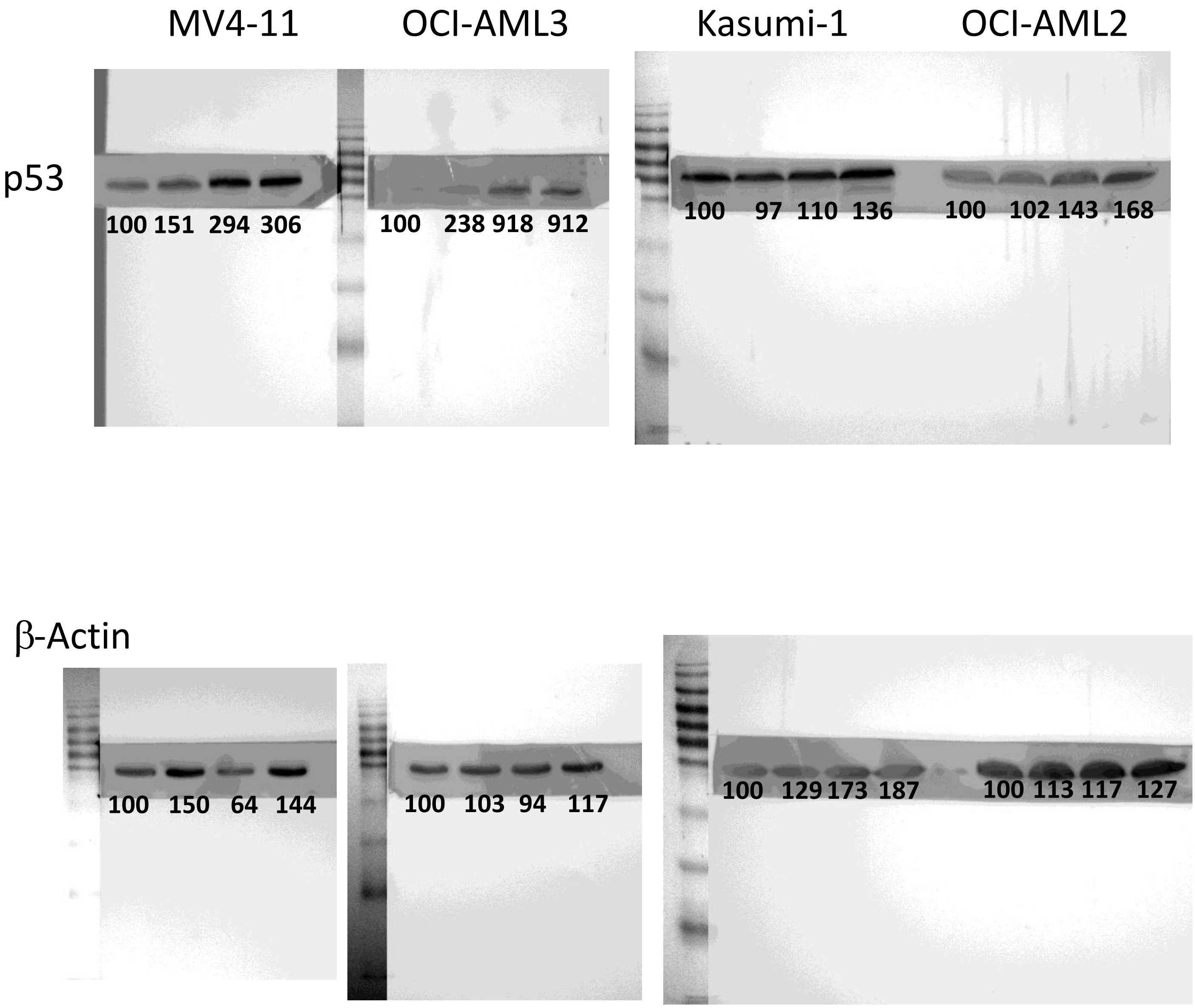
MV4-11
OCI-AML3
Kasumi-1
OCI-AML2
p53
100 151 294 306
100 238 918 912
100 97 110 136
100 102 143 168
β-Actin
100 150 64 144
100 103 94 117
100 129 173 187
100 113 117 127

Fig.11B

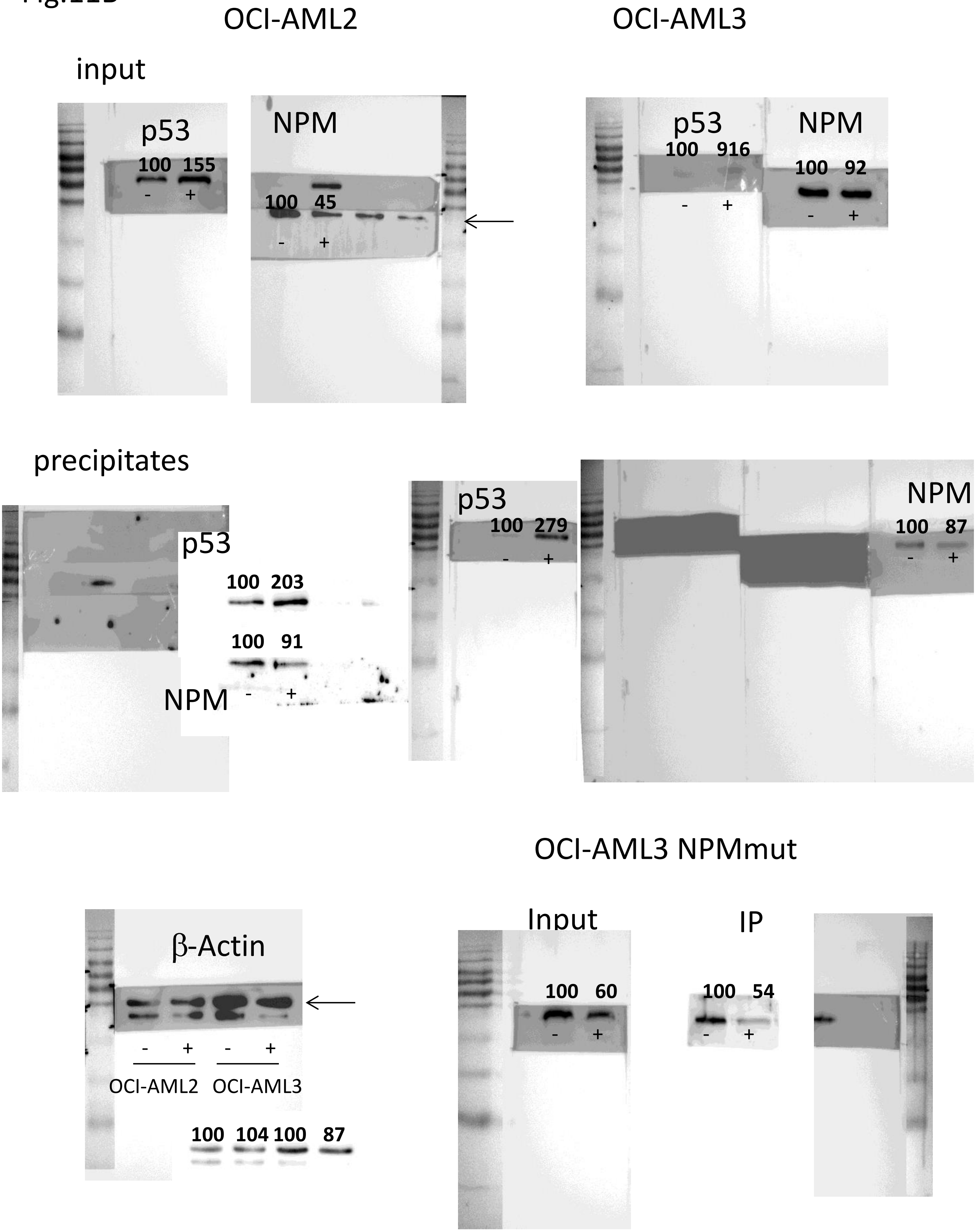
OCI-AML2
OCI-AML3
input
p53
100 155
- +
NPM
100 45
- +
p53
100 916
- +
NPM
100 92
- +
precipitates
p53
100 203
100 91
NPM - +
p53
100 279
- +
NPM
100 87
- +
OCI-AML3 NPMmut
β-Actin
- + - +
OCI-AML2 OCI-AML3
100 104 100 87
Input
IP
100 60
- +
100 54
- +

Fig.11B (continued)

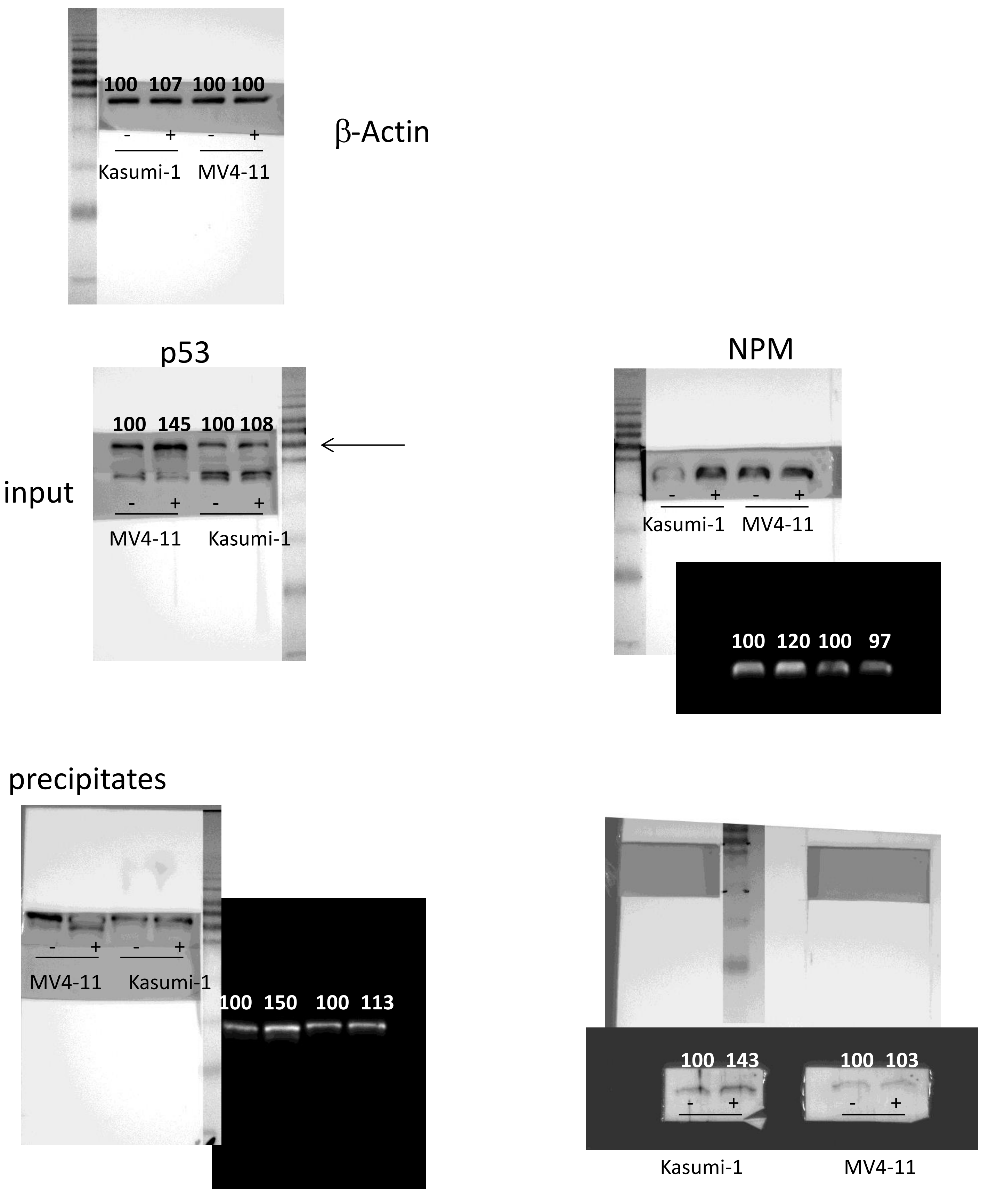

Supplement: Supplementary file 1 [file cancers-13-03266-s001.zip › File S1.pdf]
