# Supplementary material for: AML-Related NPM Mutations Drive p53 Delocalization into the Cytoplasm with Possible Impact on p53-Dependent Stress Response
Source: Cancers (Basel). 2021 Jun 29;13(13):3266. doi: 10.3390/cancers13133266 (PMC8269334; doi:10.3390/cancers13133266)
Supplement: Supplementary file 1 [file cancers-13-03266-s001.zip › Supplementary Material 1.pdf]

Supplementary material 1

AML-related NPM mutations drive p53 delocalization into the cytoplasm with possible impact on p53-dependent stress response

Holoubek A.[1], *Strachotová D.*[2], *Otevřelová P.*[1], *Röselová P.*[1], *Heřman P.*[2] *and Brodská B.*[1]

[1]Department of Proteomics, Institute of Hematology and Blood Transfusion, U Nemocnice 1, 128 20 Prague, Czech Republic
[2]Faculty of Mathematics and Physics, Institute of Physics, Charles University, Ke Karlovu 5, 121 16 Prague 2, Czech Republic

Antibody	Manufacturer	Clone, cat. No	Dilution for lysates	Dilution for co-precipitates
β-Actin	S. Cruz Biotech	C4, sc-47778	1:500	---
GFP	S. Cruz Biotech	B-2, sc-9996	1:500	1:200
dsRed	S. Cruz Biotech	E-8, sc-390909	1:500	1:100
NCL	S. Cruz Biotech	MS-3, sc-8031	1:500	1:100
FBL	S. Cruz Biotech	G-4, sc-186021	1:500	1:100
NPM total, S. Cruz	S. Cruz Biotech	3F291, sc-70392	1:500	1:200
NPM total, Abcam	Abcam	polyclonal, ab-245326	1:1000	1:500
NPMmut	Covalab	polyclonal,pab50321	1:2000	1:500
p53	Abcam	E26, ab-32389	1:2000	1:500

Table S1. Specification of primary antibodies used for protein detection in immunoblotting.

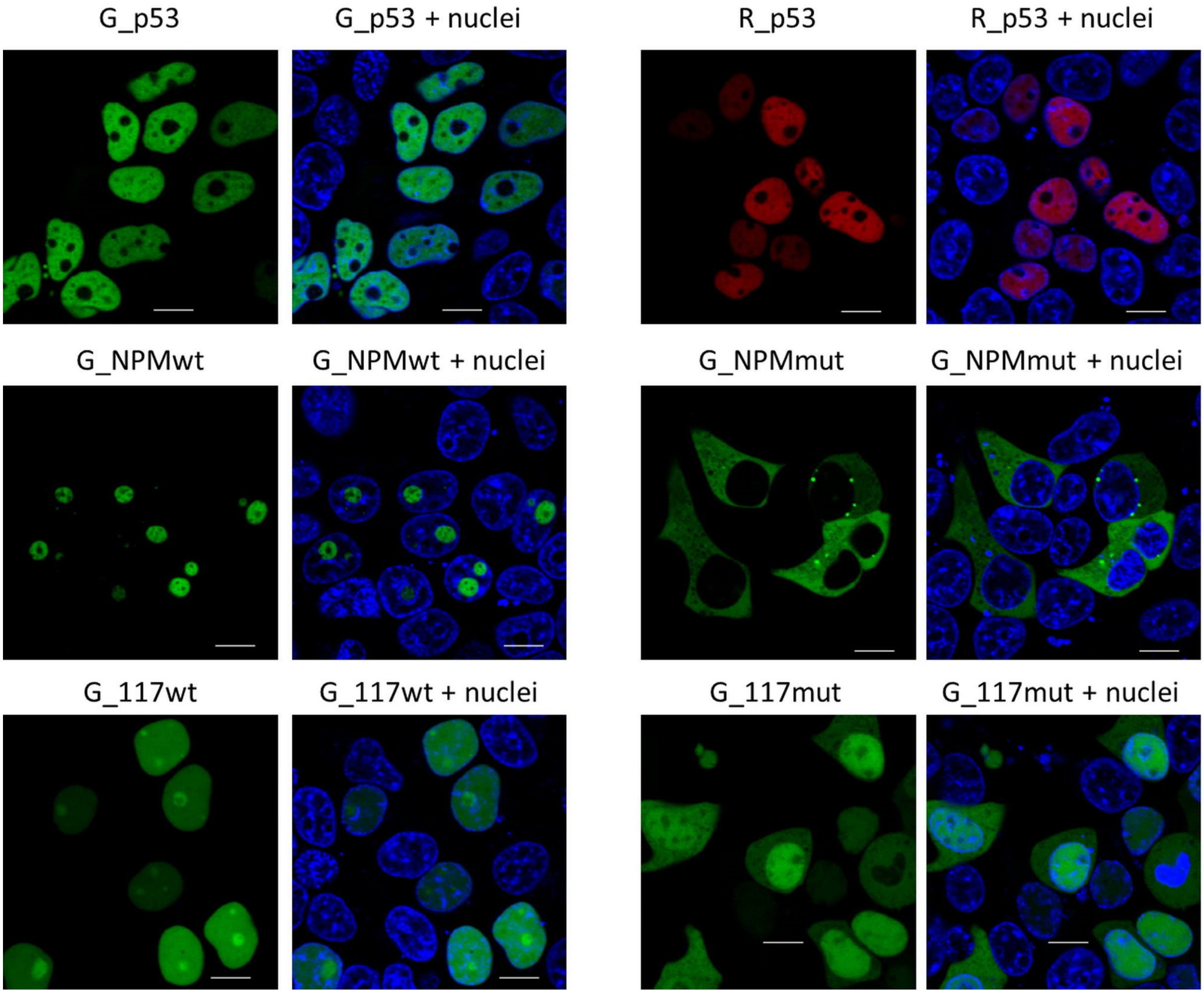

Figure S1. Localization of distinct fluorescently labeled proteins expressed in HEK-293T cells with nuclei counterstained with Hoechst33342. For p53, localization of both G_p53 and R_p53 constructs is shown to demonstrate that their cellular localization is independent of the type of the tag. The localization of NPM variants is shown only for the eGFP tag, since the mRFP1-labeled variants also localize identically. Bars represent 10 μm.

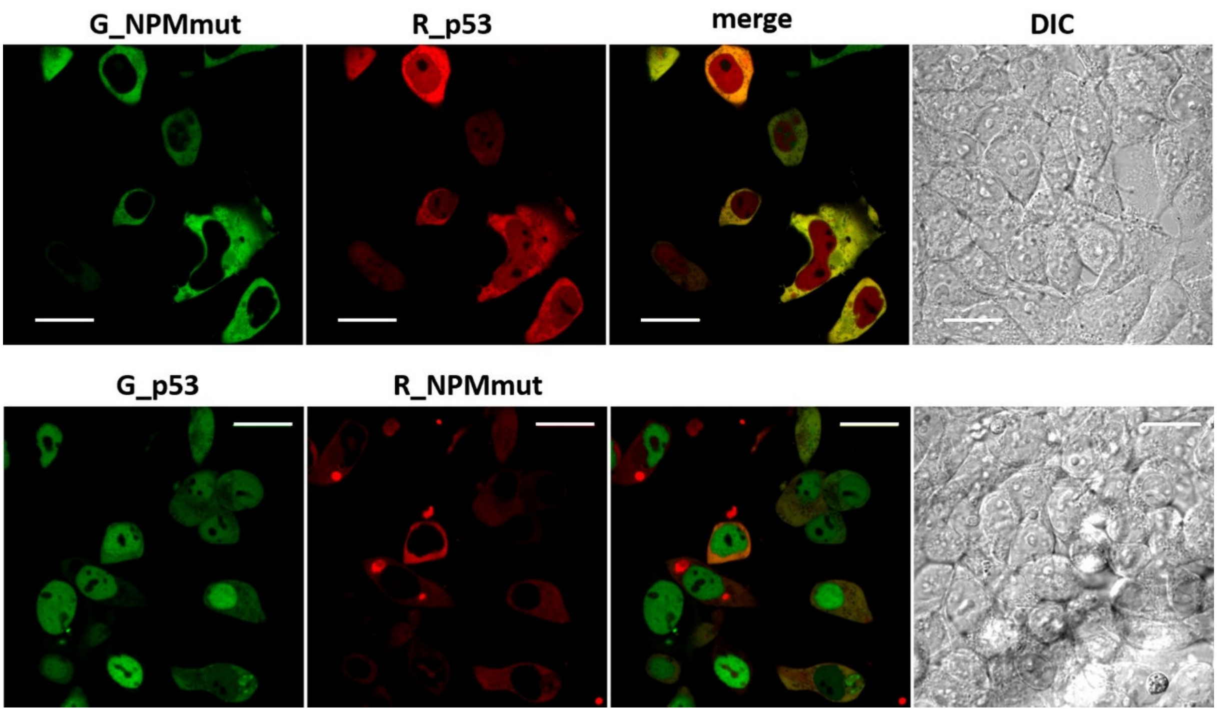

Figure S2. Localization of p53 co-expressed with NPMmut in HEK-293T cells shown for two tagging combinations. Upper row - co-expression of G_NPMmut and R_p53, lower row - co-expression of R_NPMmut and G_p53. Columns from left to right represent eGFP fluorescence, mRFP1 fluorescence, merge of both channels, and DIC image. Bars indicate 20 μm.

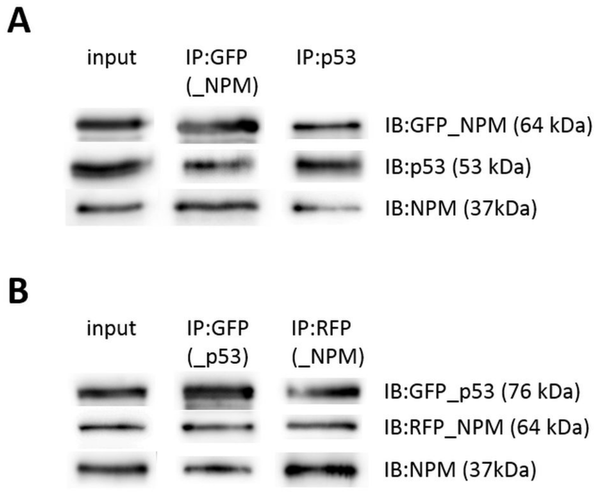

Figure S3. Interaction between NPMwt and p53 in cell lysates of transfected HEK-293T cells. (**A**) Transfection with G_NPMwt; precipitation with GFP Trap and p53-Trap. Endogenous p53 co-precipitates with NPMwt, both exo- and endogenous NPMwt co-precipitate with the p53; (**B**) Co-transfection with G_p53 and R_NPMwt; precipitation with GFP-Trap and RFP-Trap. Exogenous p53 co-precipitates with exogenous NPMwt. Both exo- and endogenous NPMwt co-precipitate with exogenous p53.

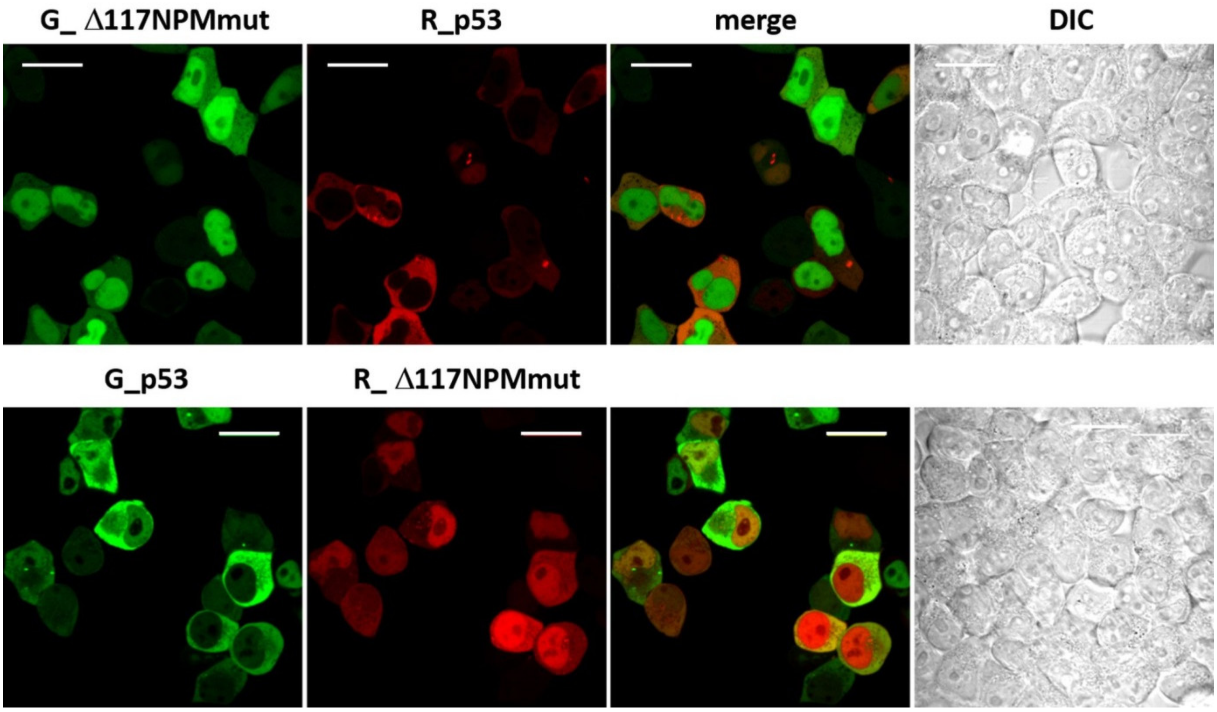

Figure S4. Localization of p53 co-expressed with Δ117mut in HEK-293T cells shown for two tagging combinations. Upper row - co-expression of G_Δ117mut with R_p53, lower row - co-expression of R_Δ117mut with G_p53. Columns from left to right represent eGFP fluorescence, mRFP1 fluorescence, merge of both channels, and DIC image. Bars indicate 20 μm.

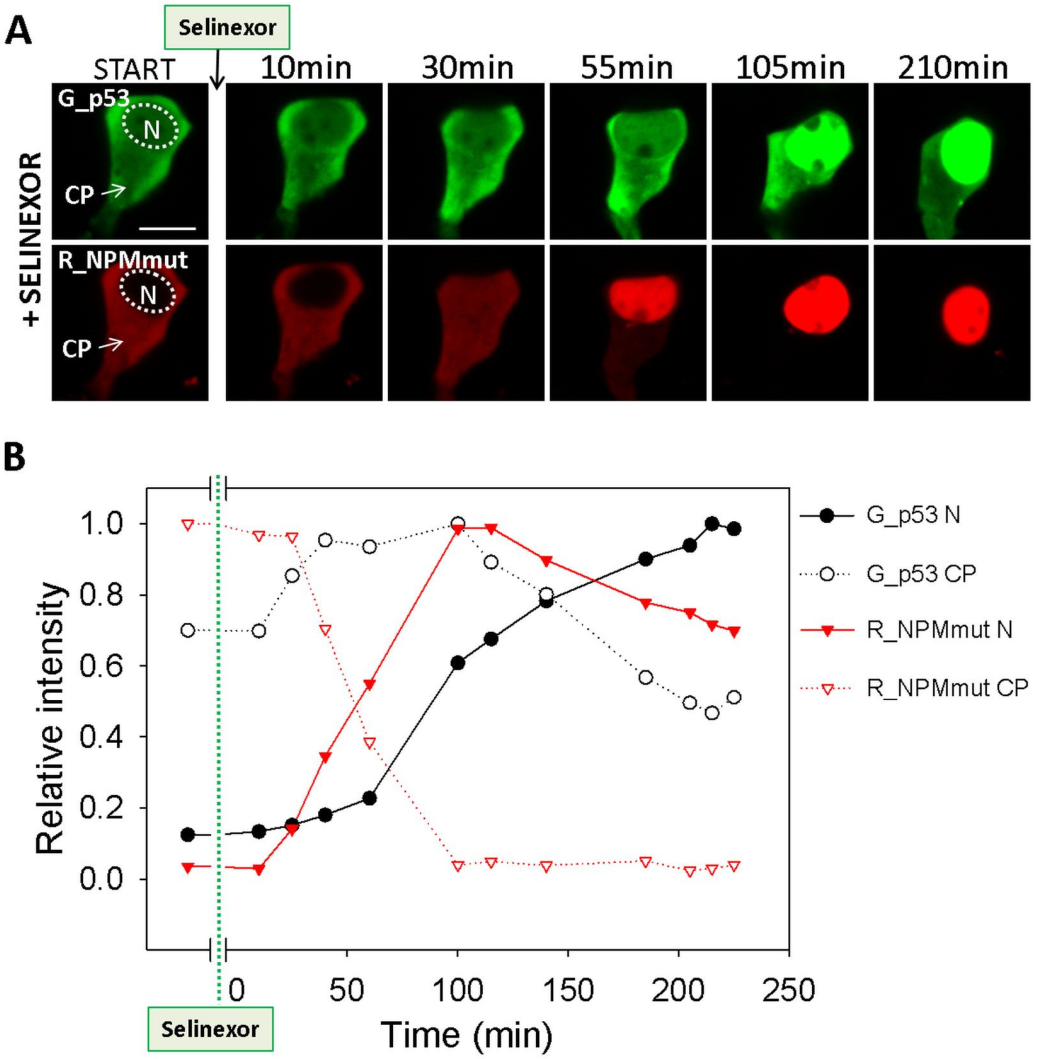

Figure S5. Effect of Selinexor on the fluorescence intensity and localization of G_p53 and R_NPMmut co-transfected in HEK-293T cells. (**A**) Intensity images of G_p53 co-expressed with R_NPMmut at different times upon the addition of 4 µM Selinexor. Depicted is the unconverted cell no. 2 from **Figure 7A**. Bar represents 10 µm; (**B**) Fluorescence intensity changes of G_p53 (circles) and R_NPMmut (triangles) in the nucleus (N) and the cytoplasm (CP) of the cell from the panel A. Areas used for integration of the fluorescence signal are separated by the dotted line in panel A. Intracellular G_p53 was not photoconverted.

Antibody	Manufacturer	Clone, cat. No	Dilution
XPO1	S. Cruz Biotech	H-7, sc-74455	1:250
PARP	S. Cruz Biotech	F-2, sc-8007	1:500
p53 pSer15	Abcam	EPR64(N), ab-223868	1:500
p53 pSer46	Abcam	EP42Y, ab-76242	1:500
p53 pSer392	Abcam	EP155Y, ab-33889	1:1000
Puma	S. Cruz Biotech	G-3, sc-374223	1:250
p21	S. Cruz Biotech	H-164, sc-756	1:250
BAX	S. Cruz Biotech	N-20, sc-493	1:500
survivin	S. Cruz Biotech	C-6, sc-374614	1:250
caspase-3	S. Cruz Biotech	E-8, sc-7272	1:500

Table S2. Specification of primary antibodies used for immunoblotting of phosphorylated p53 and apoptosis-related proteins (Figure S7)

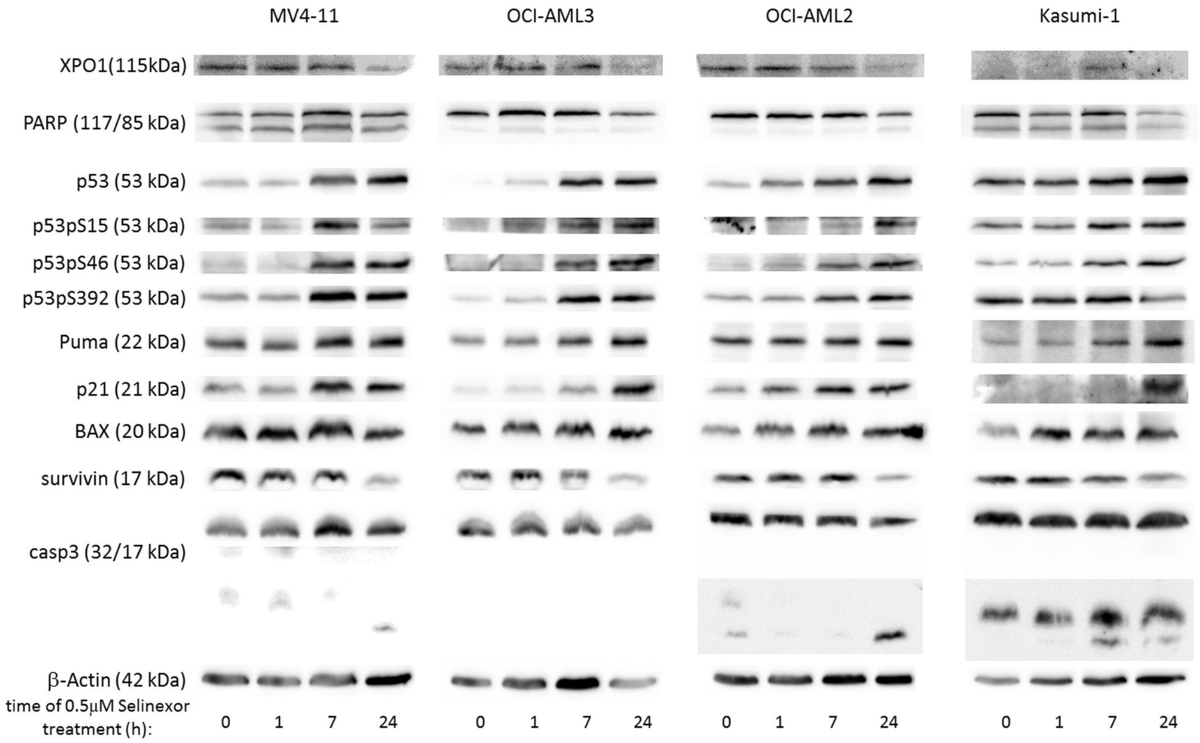

Figure S6. Expression of phosphorylated p53 variants and apoptosis-related proteins in AML cell lines after treatment with 0.5μM Selinexor. All cell lines express wild type p53 except Kasumi-1 with the R248Q mutant. Puma, p21 and BAX are pro-apototic proteins generally induced by p53, survivin is an antiapoptotic protein negatively regulated by p53. β-Actin represents a loading control. Specification of antibodies used for immunoblotting is presented in Table S2.

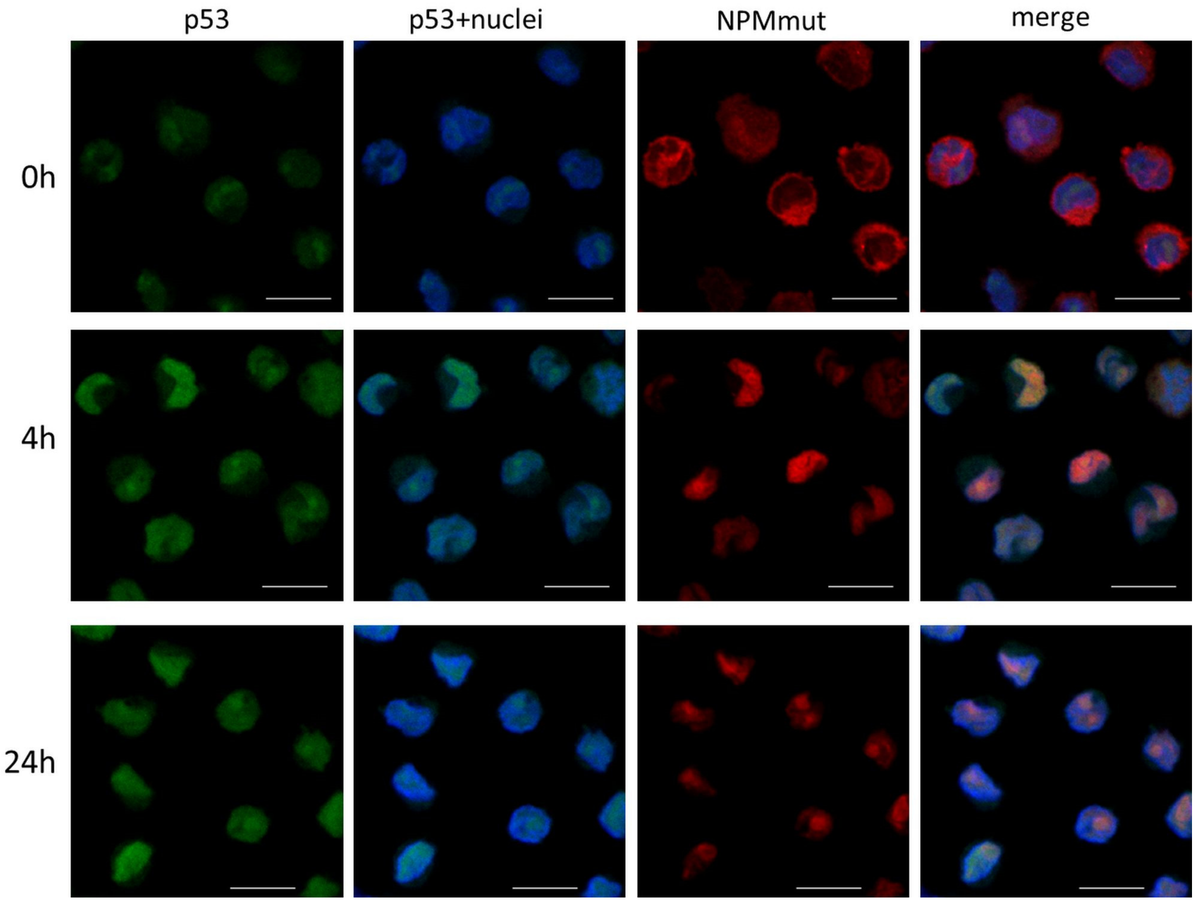

Figure S7. Localization of p53 stained with Alexa Fluor488 (green) and NPMmut stained with Alexa Fluor555 (red) in OCI-AML3 cells treated with 0.5μM Selinexor. Rows represent different times upon the Selinexor treatment. At indicated times the cells were fixed and permeabilized with PFA/Triton X-100. Nuclei were counterstained with DAPI. Bars represent 10 μm.
